# Relative contribution of muscle strength, lean mass, and lower extremity motor function in explaining between-person variance in mobility in older adults

**DOI:** 10.1186/s12877-020-01656-y

**Published:** 2020-07-28

**Authors:** Nathan P. Wages, Janet E. Simon, Leatha A. Clark, Shinichi Amano, David W. Russ, Todd M. Manini, Brian C. Clark

**Affiliations:** 1 Ohio Musculoskeletal and Neurological Institute (OMNI), Ohio University, 250 Irvine Hall, 1, Athens, OH 43147 USA; 2 Department of Biomedical Sciences, Ohio University, 250 Irvine Hall, Athens, OH 43147 USA; 3 School of Applied Health Sciences and Wellness, Ohio University, Athens, OH USA; 4 Department of Family Medicine, Ohio University, Athens, OH USA; 5 Xenoma Inc., Ota-Ku, Tokyo, Japan; 6 University of South Florida Morsani College of Medicine, School of Physical Therapy & Rehabilitation Sciences, Tampa, FL USA; 7 Department of Aging and Geriatric Research, University of Florida, Gainesville, FL USA; 8 Division of Geriatric Medicine, Ohio University, 250 Irvine Hall, Athens, OH 43147 USA

**Keywords:** Coordination, Dynapenia, Functional independence, Physical function, Sarcopenia

## Abstract

**Background:**

Approximately 35% of individuals > 70 years have mobility limitations. Historically, it was posited lean mass and muscle strength were major contributors to mobility limitations, but recent findings indicate lean mass and muscle strength only moderately explain mobility limitations. One likely reason is that lean mass and muscle strength do not necessarily incorporate measures globally reflective of motor function (defined as the ability to learn, or to demonstrate, the skillful and efficient assumption, maintenance, modification, and control of voluntary postures and movement patterns). In this study we determined the relative contribution of lean mass, muscle strength, and the four square step test, as an index of lower extremity motor function, in explaining between-participant variance in mobility tasks.

**Methods:**

In community-dwelling older adults (*N* = 89; 67% women; mean 74.9 ± 6.7 years), we quantified grip and leg extension strength, total and regional lean mass, and time to complete the four square step test. Mobility was assessed via 6-min walk gait speed, stair climb power, 5x-chair rise time, and time to complete a complex functional task. Multifactorial linear regression modeling was used to determine the relative contribution (via semi-partial r^2^) for indices of lean mass, indices of muscle strength, and the four square step test.

**Results:**

When aggregated by sex, the four square step test explained 17–34% of the variance for all mobility tasks (*p* <  0.01). Muscle strength explained ~ 12% and ~ 7% of the variance in 6-min walk gait speed and 5x-chair rise time, respectively (*p* <  0.02). Lean mass explained 32% and ~ 4% of the variance in stair climb power and complex functional task time, respectively (p <  0.02). When disaggregated by sex, lean mass was a stronger predictor of mobility in men.

**Conclusion:**

The four square step test is uniquely associated with multiple measures of mobility in older adults, suggesting lower extremity motor function is an important factor for mobility performance.

**Trial registration:**

NCT02505529–2015/07/22.

## Background

Mobility limitations, classically characterized by “slowness” when walking and/or when performing physical tasks [1–3], are highly associated with fall risk, disability, increased dependency, hospitalization, and mortality [1, 4–7], in addition to carrying an annual health care cost of ~ 42 billion in the U.S. [8]. Slow gait speed in older adults, in particular, is a strong predictor of mortality [1]. The percentage of older adults affected by mobility limitations is large, as ~ 35% of individuals > 70 years and more than 50% of individuals 85+ years currently have mobility limitation [2, 9, 10].

Previous research in older adults has focused largely on musculoskeletal mechanisms and processes underlying mobility limitations. This focus is likely due to the premise that sarcopenia, the age-related loss of lean mass [11], results in reduced muscle strength, which is a major contributor to mobility limitations in older adults [12]. However, recent findings from the Sarcopenia Definitions and Outcomes Consortium indicates the “sarcopenia variables” of lean mass and muscle strength only moderately explain limitations in mobility [13]. We postulate that lean mass and muscle strength do not fully discriminate, or predict, mobility limitations because they do not capture “motor function”. Motor function is the ability to learn, or to demonstrate, the skillful and efficient assumption, maintenance, modification, and control of voluntary postures and movement patterns [14]. We assert that motor function is an important contributor to age-related mobility limitations above and beyond the associations found with lean mass and muscle strength.

Accordingly, in the present study we sought to determine the relative contribution of muscle strength, lean mass, and an index of lower extremity motor function (performance time on the four square step test, which is discussed more below), in explaining between-participant variance in measures of mobility in older adults. To this end, we quantified various aspects of mobility (e.g., gait speed, chair rise time, stair climb power, etc.) using objectively measured, laboratory-based assessments. We chose to use the time to complete the four square step test, a multidirectional stepping test, as an index of lower extremity motor function because this test heavily challenges motor planning and initiation, as well as motor sequencing and recall, and incorporates musculoskeletal, and likely, peripheral sensory factors (i.e., strength and joint range of motion of the hip and ankle, as well as sensation/proprioception) that standard knee extensor strength and mass tests do not evaluate [15–18]. Our a priori hypothesis was the four square step test would be an independent predictor of mobility performance in community dwelling older adults. Overall, our results were consistent with this hypothesis and underscore the role of lower extremity motor function as a critical determinant of mobility in older adults.

## Methods

### General overview of the study and participants

Eighty-nine community-dwelling older adults participated in this study (Total: *N* = 89, 63–92 years, mean age 74.9 ± 6.7 years; Males: *n* = 29, 65–90 years, mean age 75.5 ± 6.3 years; Females: *n* = 60, 63–92 years, mean age 74.5 ± 6.9 years [see Table 1 for complete descriptive statistics]). To be considered for the study, participants had to be ≥60 years of age and have a body mass index (BMI) between 18 and 40 kg/m^2^. Study participants had to be living independently, and free of major musculoskeletal, neurological, cardiac, pulmonary, renal, psychiatric, and cognitive disease or disorders (see Supplementary Table 1 for a complete list of inclusion and exclusion criteria). All participants had to be willing to undergo a Dual-Energy X-ray Absorptiometry (DEXA) scan. Participants were instructed to abstain from drinking caffeinated beverages for > 4 h prior and alcohol for > 24 h prior to the testing session. The Ohio University Institutional Review Board approved this study, and all study participants had to provide written informed consent in accordance with the Declaration of Helsinki for their participation. Furthermore, this study adheres to the CONSORT guidelines. Data for this report was derived from part of a larger study/dataset (UNCODE Study; NCT02505529).

**Table 1:** Characteristics of study participants

Characteristic	Overall *N* = 89	Males *n* = 29	Females n = 60
Age (years)	74.9 ± 6.7	75.5 ± 6.3	74.5 ± 6.9
Women (%)	67.4	–	–
Height (cm)	164.3 ± 10.0	174.3 ± 7.4	159.2 ± 6.8
Weight (kg)	74.25 ± 15.9	83.7 ± 12.6	69.4 ± 15.1
BMI (kg/m^2^)	27.4 ± 5.0	27.5 ± 3.8	27.4 ± 5.5
Obesity: BMI > 30 (%)	25.8	24.1	26.7
Morbid Obesity: BMI > 35 (%)	6.7	0.0	10.0
Lean Mass (kg)	43.2 ± 10.5	54.0 ± 5.9	37.7 ± 7.6
Appendicular Lean Mass (kg)	18.3 ± 4.3	23.0 ± 2.6	15.8 ± 2.5
Appendicular Lean Mass (kg/Height^2^)	6.7 ± 1.2	7.7 ± 0.8	6.2 ± 1.0
Lean Thigh Mass (kg)	9.7 ± 2.1	11.5 ± 1.4	8.8 ± 1.8
Handgrip Strength	25.3 ± 8.4	33.3 ± 7.4	22.1 ± 5.3
Isometric Leg Extension Strength	75.7 ± 30.0	104.4 ± 27.6	60.6 ± 17.0
Isokinetic Leg Extension Strength (N-m/kg)	90.2 ± 32.6	117.6 ± 32.0	75.8 ± 21.6
SPPB Score	10.8 ± 2.1	11.2 ± 1.0	10.9 ± 1.4
Charlson Comorbidity Index Score	4.1 ± 0.9	4.1 ± 0.8	4.0 ± 1.0
Six Minute Walk Gait Speed (m/sec)	1.3 ± 0.3	1.4 ± 0.2	1.3 ± 0.3
Moderate-Vigorous Activity, min/week	118.4 ± 60.5	117.3 ± 53.6	114.8 ± 65.9
RBANS Score	106.6 ± 12.2	102.2 ± 13.0	108.9 ± 11.5

BMI = body mass index; N = Newtons; m = meters; RBANS = Repeatable Battery for the Assessment of Neuropsychological Status; sec = second; SPPB = Short Physical Performance Battery

With respect to data pertinent to this report, we quantified the following outcomes: total and regional lean mass via DEXA, grip strength, isometric and isokinetic (60°/sec) leg extension strength, and time to complete the four square step test. We also quantified various aspects of mobility using objectively measured, laboratory-based assessments. Here, participants completed two locomotor (6-min walk gait speed and stair climb power) and two non-locomotor (5x chair rise time and time to complete a complex functional task) laboratory-based tests. To permit characterization of our study participants, we also quantified short physical performance battery test score [19, 20], moderate-vigorous intensity physical activity via accelerometry [21], neuropsychological status via the Repeatable Battery for the Assessment of Neuropsychological Status (RBANS [22];) and comorbidities via the Charlson Comorbidity Index [23]. Below we describe the methodological details related to our primary variables of interest.

### Lean tissue mass

DEXA scans (Hologic Discovery QDR model Series, Waltham, MA, USA) were performed to assess lean mass as per our prior description [24]. A whole-body scan was performed, and whole-body, appendicular, and lower extremity lean tissue mass was determined using the system’s software package (Hologic APEX, Version 4.0.2). Participants were advised to report to the laboratory in a hydrated state and were given scrubs to wear during the scan. Care was taken to follow The International Society for Clinical Densitometry guidelines for positioning during the scan [25].

### Muscle strength

Isometric and isokinetic leg extension maximal voluntary contraction (MVC) strength measures were recorded utilizing a Biodex System 4 Dynamometer (Biodex Medical Systems Inc., Shirley, NY). For quantification of isometric MVC strength, participants were seated with their non-dominant leg at 90° flexion and the knee axis of rotation in alignment with the rotational axis of the Biodex torque motor. A lap belt was applied to prevent movement at the hip, and the participant’s non-dominant lower extremity was affixed to a lower extremity lever arm, which was attached to the Biodex torque motor. The torque signal was scaled to maximize its resolution (208.7 mV/Nm; Biodex Researchers Tool Kit Software) and sampled at 625 HZ (MP150 Biopac Systems). Participants received visual feedback of forces on a monitor located 1-m in front of them. Provided with strong verbal encouragement, participants performed three isometric MVCs with 1 min of rest between each effort, and the peak value of these three trials was utilized as the isometric MVC for the analysis.

Isokinetic MVC strength was conducted on the same leg, using the same general set-up, but isokinetic concentric torque was measured at 60°/sec. Six isokinetic trials were performed with 30 s rest between bouts. Isokinetic MVC strength was calculated as the mean of the highest three values of maximal isokinetic torque produced between 90° and 30° of leg flexion. This isokinetic MVC strength testing protocol corresponds to that used in the Health ABC study, which established cut-points for future development of severe mobility [26].

Grip strength was assessed in a manner similar to that described by Villanfañe (2015) [27] using a portable JAMAR® Hydraulic Hand Dynamometer; (Model 5030 J1; Lafayette Instrument Co.; Lafayette, Indiana). In short, after demonstrating its use, the research staff measured the participant’s hand and fingers in the dynamometer to appropriately set up the handle position. The test was performed in the upright seated position with the shoulder of the tested arm adducted to the side, the elbow flexed at 90°, and the forearm and wrist aligned in the neutral position. Next, the dynamometer was placed in the participant’s hand and they were asked to squeeze the dynamometer as hard as possible for 3 s and then let go. Trials were performed with 30 s rest between bouts and measurements were recorded to the nearest 0.5 kg. This process was conducted three times per hand, and the dynamometer was set to the zero kg mark before each measurement. If the relative difference between trials was within 10%, no additional trials were required. If not, additional trials were performed until three trials were within 10%. Once completed, the average of the highest 3 trials were recorded for each hand.

### Four square step test

The four square step test was conducted in a manner similar to that described by Dite and Temple (2002) [15], except we asked participants to step over tape instead of canes. Briefly, participants were required to step in a predetermined sequence over four 76-cm-long pieces of white tape, placed in a cross configuration on the ground over dark colored carpet. Participants were instructed to complete the sequence as fast as possible without touching the pieces of white tape and time to task completion was recorded. The trial was considered a failure and repeated if the sequence was not completed correctly, the participant lost their balance, or if a foot touched the tape line. Trials were only repeated if the participant failed and each participant performed 3 trials with 30 s rest between trials. Test scores were taken from the mean of trials 1–3 and the task was measured to the nearest 0.01 s using a stopwatch.

#### Six-minute walk distance

Participants were instructed to walk as quick and as far as possible in 6 min (on a 60-m course). Specifically, participants were instructed to walk in a straight line for 30 m, complete a 180° turn to the left around a cone and walk back to the starting line, which was an additional 30 m. Once participants arrived at the starting line, they completed another 180° turn to the left around an additional cone and proceeded to repeat this sequence as many times as possible. Markers were set every 3 m to accurately determine the distance completed in 6 min. Participants were told the remaining time at each turn and verbal encouragement was provided. This task was performed once.

#### Stair climb

Stair climb power was calculated as: power = force x velocity, where force = body mass in kilograms x acceleration due to gravity, and velocity = cumulative stair height/stair climb time. Prior to the start of this test, participants were weighed to the nearest pound, and were instructed to stand at the bottom of a flight of eight stairs (each stair is 7 in. in height). Next, participants were instructed to climb a flight of stairs as quickly as possible and told not to use the handrail unless they were unable to climb the stairs without it. The time required to complete the test was measured using switch mats (Lafayette Instruments Model 63516A) interfaced with a digital timer (Lafayette Instruments Model 54,060). The task was performed twice, was measured to the nearest 0.01 s, and the results were averaged between trials.

#### 5x chair rise

Participants were asked to sit in a chair with a pan height of ~ 45 cms (height includes ~ 4 cms of padding). Participants were instructed to cross their arms, place their hands on the opposite shoulder, and starting from an upright seated position, they were instructed to stand up and then sit back down five times consecutively keeping both feet on the floor throughout the test. The task was performed once and was measured to the nearest 0.01 s using a stopwatch.

#### Complex functional task

Participants were asked to sit upright on the floor with their legs extended in front of them with their knees bent to ~ 45°. A laundry basket was positioned in front of their feet. Participants were permitted to place their hands on the ground behind themselves if needed or preferred. Starting from the seated position, participants were instructed to transition from sitting on the floor to standing, then lift the laundry basket that weighed a total of 4.5 kg, walk 1.5 m, and place the basket on a 0.75-m high table. For participants who were unable to complete the task or those who took > 30 s to complete the task, a time value of 30 s was assigned. The task was performed twice, was measured to the nearest 0.01 s using a stopwatch, and the results were averaged between trials. If the participant was unable to perform the test a second time, the first attempt was used to represent their value.

### Statistical analysis

The relative contribution of muscle strength, lean mass, and the four square step test (representing the lower extremity motor function construct) in explaining the between-participant variance in measures of mobility (i.e., six-minute walk gait speed, stair climb power, 5x chair rise time, and time to complete a complex functional task) in older adults was assessed via multifactorial linear regression analyses. Independent predictor variables were muscle strength, lean mass, and the four square step test. Notably, measures of muscle strength and lean mass were expressed in a variety of forms so as to maximize the opportunity for these constructs to contribute to the respective models (see Supplementary Table 2 for a complete list). Furthermore, these three variables did not demonstrate collinearity (i.e., none of the predictor variables had associations ≥0.60). Additionally, the variance inflation factor (VIF) was computed to evaluate multicollinearity for each regression model. Note that VIF values under 2.5 are considered acceptable [28].

To evaluate the independent contribution of each predictor variable, we calculated the semi-partial r^2^ value (_sp_r^2^) as it is interpreted as the variance uniquely attributed to a given predictor variable by factoring out the shared variance from all other variables [29]. We recognize that the relative contribution of the constructs of lean mass and muscle strength could vary depending on how it is expressed (e.g., an absolute value vs. a value relative to body stature), as well as various nuances of what is being measured (e.g., grip strength vs. isometric leg extensor strength vs. isokinetic leg extensor strength; upper thigh lean mass vs. appendicular lean mass, etc.). Thus, in our analyses we expressed lean mass and muscle strength constructs several different ways (see Supplementary Table 2 for a complete list).

The regression models presented herein are those with the “best” model fit characteristics, not necessarily the ones with the “best” independent contribution of the predictor variables. To determine the model of best fit for each dependent variable, we independently evaluated the Schwarz Bayesian Criterion (SBC) [30] and the Akaike’s Information Criterion (AIC) [31] for goodness-of-fit for each individual model and not across models. The models with the “best” fit (i.e., closer to 0, or even below 0) are presented in the results. Statistical Package for Social Sciences (SPSS) version 25 (SPSS Inc., Chicago, IL) was used for all statistical analyses, all data was expressed as mean ± SD for descriptive statistics, and all statistical tests were set at the 5% significance level (2-tailed).

If data was missing for a participant, we used an imputation statistical software package from SPSS to run a Markov Chain Monte Carlo algorithm, known as fully conditional specification or chained equations imputation. The Markov Chain Monte Carlo method was used because multivariate normality was acceptable [32]. The algorithm imputes incomplete variables one at a time, using the filled-in variables as a “pooled output” to estimate what the value would have been if the original dataset had no missing values [33]. The imputation software was only used when 10% or less of the population were missing values for a particular variable. Thus, we could only allow missing values for up to eight participants for any given variable. Notably, no variables had missing values > 10% of the study population. In addition, we conducted a sensitivity analysis with and without the missing values and found there no statistical difference.

#### Power analysis

Post-hoc estimation of statistical power and effect size was determined for the overall model using the smallest R^2^ value of 0.5 (5x chair rise model), an α = 0.05, a *n* = 89, and three predictors, which resulted in a post-hoc power of 0.99. For the male-specific model, post-hoc estimation of power was based on the smallest R^2^ value of 0.29 (CFT model), an α = 0.05, a *n* = 23, and three predictors, which resulted in a post-hoc power of 0.73. Lastly, for the female-specific model, post-hoc estimation of power was based on the smallest R^2^ value of 0.42 (stair climb power model), an α = 0.05, a *n* = 60, and three predictors, which resulted in a post-hoc power of 0.99. All post-hoc power calculations were conducted using G*Power 3.1 [34].

## Results

### Overall model results (Table 2)

**Fig. 1:**
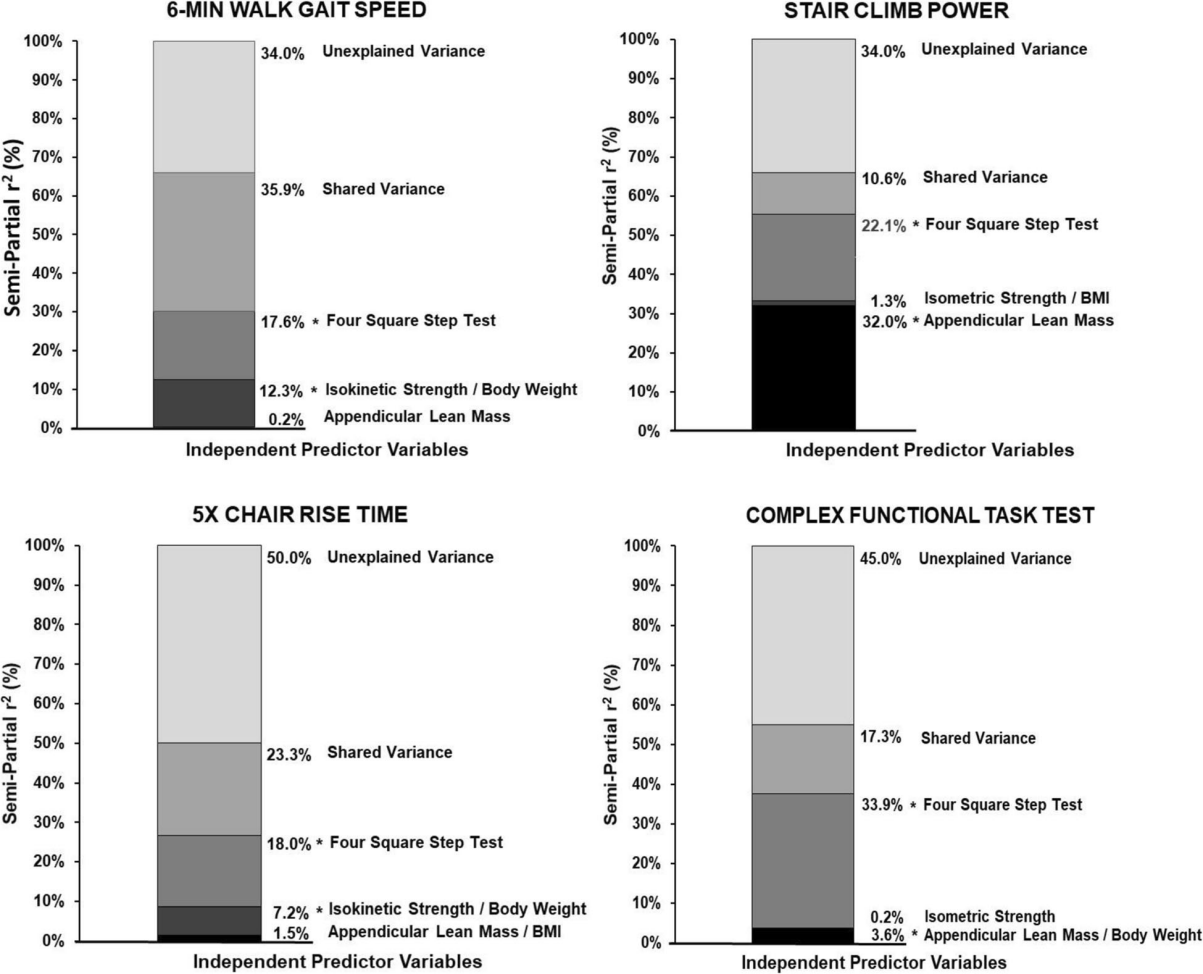
Relative contribution of the constructs of lean mass, muscle strength, and lower extremity motor function (assessed via the four square step test) on 6 min walk gait speed (A), stair climb power (B), 5x chair rise time (C), and time to complete the complex functional task (D). An asterisks symbol (*) implies that a predictive variable was significant

The results when both men and women were included in the models are displayed in Table 2. The overall R^2^ values explained by the four square step test, muscle strength, and lean mass, for 6-min walk gait speed, stair climb power, 5x chair rise time, and the time to complete the complex function task, were 0.66, 0.66, 0.50, and 0.53, respectively (*p* <  0.01). The four square step test uniquely explained 17–34% of the between subject variance for all the mobility tasks (i.e., _sp_r^2^ values = 0.17–0.34; p <  0.01) (Fig. 1). Muscle strength uniquely explained ~ 12% and ~ 7% of the between-participant variability in 6-min walk gait speed and chair rise time, respectively (*p* <  0.02) (Fig. 1). Lean mass uniquely explained 32% and ~ 4% of the between-participant variance in stair climb power and the time to complete the complex functional task (p <  0.02) (Fig. 1). In addition, the VIF values for each predictor variable are acceptable with all values under 2.2.

**Table 2:** Overall model summary

VARIABLES		MODEL CHARACTERISTICS		
**DEPENDENT**	**INDEPENDENT**	**s-p r**^**2**^	**VIF**	***p*-value**
6-MIN WALK GAIT SPEED	App. Lean Mass	0.002	1.072	0.523
Overall Model	Isokin. Strength / BW	0.123	1.450	< 0.001*
R^2^ = 0.66; p-value = < 0.001*	Four Sq. Step Test	0.176	1.383	< 0.001*
AIC = 673.3; MPC = 4.000	Shared Variance	0.359	–	–
BIC = 682.8; D-W = 2.000	Unexplained Variance	0.340	–	–
STAIR CLIMB POWER	App. Lean Mass	0.300	1.427	< 0.001*
Overall Model	Isomet. Strength / BMI	0.010	1.687	0.140
R^2^ = 0.66; p-value = < 0.001*	Four Sq. Step Test	0.214	1.221	< 0.001*
AIC = 544.7; MPC = 4.000	Shared Variance	0.136	–	–
BIC = 554.2; D-W = 1.564	Unexplained Variance	0.340	–	–
5x CHAIR RISE	App. Lean Mass / BMI	0.015	1.746	0.130
Overall Model	Isokin. Strength / BW	0.072	2.147	0.001*
R^2^ = 0.50; p-value = < 0.001*	Four Sq. Step Test	0.180	1.383	< 0.001*
AIC = 193.6; MPC = 4.000	Shared Variance	0.233	–	–
BIC = 203.2; D-W = 1.995	Unexplained Variance	0.500	–	–
COMPLEX FUNCTIONAL TEST	App. Lean Mass / BW	0.036	1.433	0.018*
Overall Model	Isokin. Strength	0.002	1.568	0.582
R^2^ = 0.53; p-value = < 0.001*	Four Sq. Step Test	0.339	1.237	< 0.001*
AIC = 310.5; MPC = 4.000 BIC = 320.1; D-W = 2.220	Shared Variance	0.153	–	–
	Unexplained Variance	0.470	–	–

AIC = akaike info criterion; App = appendicular; BMI = body mass index; BW = body weight; CFT = complex functional task; Dep. = dependent; DIST. = Distance; D-W = Durbin-Watson; Ht = height; Isokin = isokinetic; Isomet. = isometric; Min = minute; MPC = mallows’ prediction criterion; Pred. = Predictor; SBC = schwarz bayesian criterion; s-p = semi-partial; sq. = square; var. = variable; VIF = variance inflation factor. * = significance (*p* <  0.050)

### Men only model results (Table 3)

The results when only men were included in the models are displayed in Table 3. The overall R^2^ values explained by the four square step test, muscle strength, and lean mass, for 6-min walk gait speed, stair climb power, 5x chair rise time, and the time to complete the complex function task, were 0.47, 0.55, 0.52, and 0.29, respectively (*p* <  0.05). Lean mass uniquely explained 23–31% of the between-participant variance for 6-min walk gait speed, stair climb power, and the time to complete the complex function task (*p* <  0.01). Muscle strength uniquely explained 40% of the between-participant variance for the chair rise time (p <  0.01). The four square step test uniquely explained ~ 12% of the between-participant variance for stair climb power (*p* <  0.01). In addition, the VIF values for each predictor variable are acceptable with all values under 1.6.

**Table 3:** Model summary for sex (male)

VARIABLES		MODEL CHARACTERISTICS		
DEPENDENT	INDEPENDENT	s-p r^2^	VIF	*p*-value
6-MIN WALK GAIT SPEED	App. Lean Mass / BW	0.184	1.516	0.011*
Overall Model	Isomet. Strength / BMI	0.000	1.491	0.898
R^2^ = 0.45; p-value = 0.003*	Four Sq. Step Test	0.082	1.131	0.076
AIC = 229.4; MPC = 4.000	Shared Variance	0.184	–	–
BIC = 234.5; D-W = 1.735	Unexplained Variance	0.550	–	–
STAIR CLIMB POWER	Lower Limb Lean Mass	0.304	1.151	< 0.001*
Overall Model	Isokin. Strength / BMI	0.044	1.314	0.146
R^2^ = 0.55; p-value = < 0.001*	Four Sq. Step Test	0.118	1.154	0.007*
AIC = 192.1; MPC = 4.000	Shared Variance	0.084	–	–
BIC = 197.3; D-W = 2.047	Unexplained Variance	0.450	–	–
5x CHAIR RISE	App. Lean Mass / Ht^2^	0.046	1.000	0.121
Overall Model	Isomet. Strength / BW	0.470	1.091	< 0.001*
R^2^ = 0.59; p-value = < 0.001*	Four Sq. Step Test	0.001	1.091	0.572
AIC = 34.9; MPC = 4.000	Shared Variance	0.073	–	–
BIC = 40.08; D-W = 1.699	Unexplained Variance	0.410	–	–
COMPLEX FUNCTIONAL TEST	App. Lean Mass / BW	0.266	1.193	0.007*
Overall Model	Isokin. Strength	0.016	1.167	0.475
R^2^ = 0.29; p-value = 0.046*	Four Sq. Step Test	0.000	1.151	0.951
AIC = 109.7; MPC = 4.000	Shared Variance	0.008	–	–
BIC = 114.9; D-W = 1.822	Unexplained Variance	0.710	–	–

AIC = akaike info criterion; App = appendicular; BMI = body mass index; BW = body weight; CFT = complex functional task; Dep. = dependent; DIST. = Distance; D-W = Durbin-Watson; Ht = height; Isokin = isokinetic; Isomet. = isometric; Min = minute; MPC = mallows’ prediction criterion; Pred. = Predictor; SBC = schwarz bayesian criterion; s-p = semi-partial; sq. = square; var. = variable; VIF = variance inflation factor. * = significance (p <  0.050)

### Women only model results (Table 4)

The results when only women were included in the models are displayed in Table 4. The overall R^2^ values explained by the four square step test, muscle strength, and lean mass, for 6-min walk gait speed, stair climb power, 5x chair rise time, and the complex function task test, were 0.76, 0.42, 0.55, and 0.80, respectively (*p* <  0.01). The four square step test uniquely explained between 11 and 29% of the between-participant variance for all the mobility tasks (p <  0.01). Muscle strength uniquely explained 4–17% of the between-participant variance for 6-min walk gait speed, 5x chair rise time, and the complex function task test, respectively (p <  0.01). Lean mass uniquely explained 15% of the between-participant variance for the time to complete the complex functional test (p <  0.01). In addition, the VIF values for each predictor variable are acceptable with all values under 2.2.

**Table 4:** Model summary for sex (female)

VARIABLES		MODEL CHARACTERISTICS		
DEPENDENT	INDEPENDENT	s-p r^2^	VIF	*p*-value
6-MIN WALK GAIT SPEED	App. Lean Mass	0.013	1.049	0.110
Overall Model	Isokin. Strength / BMI	0.169	1.705	< 0.001*
R^2^ = 0.76; p-value = < 0.001*	Four Sq. Step Test	0.106	1.702	< 0.001*
AIC = 437.8; MPC = 4.000	Shared Variance	0.472	–	–
BIC = 445.7; D-W = 2.137	Unexplained Variance	0.240	–	–
STAIR CLIMB POWER	App. Lean Mass / BMI	0.005	1.680	0.517
Overall Model	Isokin. Strength / BW	0.000	2.098	0.833
R^2^ = 0.42; p-value = < 0.001*	Four Sq. Step Test	0.282	1.588	< 0.001*
AIC = 378.9; MPC = 4.000	Shared Variance	0.133	–	–
BIC = 386.7; D-W = 1.662	Unexplained Variance	0.580	–	–
5x CHAIR RISE	App. Lean Mass / BMI	0.014	1.680	0.404
Overall Model	Isokin. Strength / BW	0.134	2.098	0.008*
R^2^ = 0.55; p-value = < 0.001*	Four Sq. Step Test	0.265	1.588	< 0.001*
AIC = 141.6; MPC = 4.000	Shared Variance	0.137	–	–
BIC = 149.4; D-W = 2.114	Unexplained Variance	0.450	–	–
COMPLEX FUNCTIONAL TEST	App. Lean Mass	0.150	1.228	< 0.001*
Overall Model	Handgrip Strength	0.035	1.932	0.005*
R^2^ = 0.80; p-value = < 0.001*	Four Sq. Step Test	0.283	1.654	< 0.001*
AIC = 172.2; MPC = 4.000	Shared Variance	0.332	–	–
BIC = 180.2; D-W = 1.895	Unexplained Variance	0.200	–	–

AIC = akaike info criterion; App = appendicular; BMI = body mass index; BW = body weight; CFT = complex functional task; Dep. = dependent; DIST. = Distance; D-W = Durbin-Watson; Ht = height; Isokin = isokinetic; Isomet. = isometric; Min = minute; MPC = mallows’ prediction criterion; Pred. = Predictor; SBC = schwarz bayesian criterion; s-p = semi-partial; sq. = square; var. = variable; VIF = variance inflation factor. * = significance (*p* < 0.050)

## Discussion

In this study, we examined the relative contribution of the constructs of muscle strength and lean mass, when compared to the four square step test (as index of lower extremity motor function), in explaining between-participant variance in measures of mobility in older adults. Our a priori hypothesis was the four square step test would be an independent predictor of mobility performance in community-dwelling older adults. Overall, our results are consistent with this hypothesis and underscore the role of lower extremity motor function as a factor in mobility among older adults. Specifically, when data were aggregated by sex, the four square step test was the strongest predictor variable for all the functional tasks, except stair climb power, where the predictor variable representing lean mass (i.e., appendicular lean mass) had the highest contribution. Interestingly, when data were disaggregated by sex, we found that for men, the predictor variables representing lean mass were the strongest contributors, except for 5x chair rise time, where the predictor variable representing muscle strength (i.e., isometric strength by body weight) had the highest contribution. We should note upfront that we were underpowered for sex-specific comparisons, as we had 89 participants with only 29 males, and our predictor variables contained a fair amount of shared variance in some models. As such, our sex-specific findings should be interpreted with caution. In addition, a low _sp_r^2^ in a given variable should not be interpreted to suggest that it was not an important determinant of mobility, per se, but rather that it did not *uniquely* explain the between-participant variance. Below we further discuss these findings.

As noted in the introduction, prior research on mobility limitation in older adults has focused largely on factors related to musculoskeletal mechanisms and processes, namely the role of lean mass and muscle strength [35–40]. Indeed, numerous studies demonstrate links between the amount of lean mass, muscle strength, and mobility/physical function. For instance, Janssen et al. (2002) [35] sought to test the hypothesis that low lean mass is related to functional impairment and physical disability in older adults. These authors reported an increased likelihood of functional impairment in older men and women with a reduction in lean mass of 31 and 22%, respectively. Visser and colleagues (2005) [36] investigated the independent and joint contributions of low lean mass, low muscle strength, and greater fat infiltration into the muscle on incident mobility limitations in older adults. These authors found an association between low lean mass (smaller cross-sectional muscle area) and functional decline, which was a function of underlying muscle strength. Similarly, Reid et al. (2008) [37] reported lower extremity lean mass to be an important determinant of physical performance among functionally-limited elderly adults. In addition, Curcio et al. (2016) [38] investigated the relationship between lean mass, muscle strength, and a composite measure of mobility (Tinetti Mobility Test) in non-institutionalized older adults. These authors found a significant linear relationship between lean mass (*r* = 0.61; measured via bioelectrical impedance analysis [BIA]) and scores on the Tinetti mobility test, as well as between relative strength (*r* = 0.53; determined via grip strength by BMI) and scores on the Tinetti mobility test. However, recent meta-analyses and studies pooling data across several large epidemiological studies indicate the contribution of muscle mass to mobility appears to be considerably less than originally postulated [13, 39]. For instance, Schapp et al. (2013) [39], conducted a meta-analysis of 50 studies and noted that lower muscle strength, as well as a high BMI (> 30), were associated with functional decline, but low lean mass was not significantly associated with functional decline. Most recently, the Sarcopenia Definitions and Outcomes Consortium group, which assembled data from eight Epidemiological Cohorts (*n* = 18,831), reported that grip strength (expressed in absolute terms and relative to BMI) was a discriminator of risk for mobility disability (walking speed < 0.8 m/s), but that DEXA-derived lean mass measures were not good discriminators of mobility disability [13]. Our findings extend this prior work and suggest the contribution of lean mass and muscle strength vary depending on the task, in addition to likelihood of varying by sex.

We also interpret our findings to suggest that one reason the current sarcopenia variables of mass and strength do not adequately discriminate, or predict, mobility limitations as they are insufficient to explain “motor function”. Safe and effective negotiation of complex environments requires the integration of sensory information with neural networks, which involves cortical, subcortical, brainstem, and spinal cord structures [41]. Considering the multifactorial nature of mobility, numerous neural substrates and circuits are implicated in age-related mobility declines [42]. We contend that the reason the four square step test is a unique determinant of mobility capacity in our study is because it heavily challenges motor planning and initiation, as well as motor sequencing and recall, and incorporates musculoskeletal, and likely, peripheral sensory factors that standard knee extensor strength and mass tests do not evaluate. Choice stepping reaction time tests, such as the four square step test, have been shown to discriminate older adults who have fallen, from those who have not [15, 43, 44]. Within this context, we should further note that the four square step test alone demonstrated an area under the receiver operating curve of 0.83 for discriminating those whose gait speed was ≤0.80 m/sec during the 6 min walk test, in comparison to those whose gait speed was > 0.80 m/sec (data not shown in the results due to the small sample size).

The four square step test has been used in the study of a number of neurological diseases (e.g., Parkinson’s disease, multiple sclerosis, and cerebral palsy), as it demands complex anticipatory postural adjustments for gait initiation, known to be impaired with these populations [45–47]. However, it has not been widely used in the fields of aging systems and geriatrics. Considering that the test is clinically viable (i.e., it is quick [2–3 min], cheap, and easy to perform), we encourage future investigations, particularly the large cohort epidemiological studies, to incorporate this measure to examine whether it increases the sensitivity and specificity of the current sarcopenia variables of mass and strength in discriminating and predicting mobility limitations in older adults.

As previously mentioned, the relative contribution of the various predictor variables differed depending on the task in question. We purposely chose tasks that measured different aspects of mobility, including locomotor and non-locomotor tasks. As such, it is not surprising that the relative contribution varied by task. Again, we caution about interpretation as this should not be taken to suggest that a given variable is not an important determinant for measure of mobility, per se, but rather that it did not *uniquely* explain the between-participant variance. We should also note the relative contribution of the predictors varied by sex and task. This raises the question of: Do the determinants for measures of mobility limitations differ between sex? Our sample size is too small to definitively answer this question, but our findings certainly suggest that future work along this line of questioning is warranted.

There are limitations of this work that should be acknowledged. First, there could be better methods and/or approaches to assay motor function, strength and/or mass, as well as assessing measures of mobility. To counter this limitation, we expressed our lean mass and muscle strength data in a variety of ways to maximize their opportunity for these constructs to contribute to the respective models. Within this context we should also note that we, and others, have recently questioned the use of DEXA for assessing muscle mass over time [24, 48, 49]. However, DEXA is tightly correlated with gold standard measures of mass via magnetic resonance imaging (MRI) and computerized tomography (CT) scans cross-sectionally [24, 50–52]. Second, our sample size is relatively small for a study of this nature. Moreover, our sample size of men was roughly half that of women. Thus, our sex-specific results are underpowered and should be interpreted with caution. Lastly, we should note that this was a cross-sectional study design, which has obvious limitations in comparison to longitudinal studies.

## Conclusions

We sought to determine the relative contribution for indices of muscle strength and lean mass, and lower extremity motor function in explaining between-participant variance in measures of mobility in older adults. Our a priori hypothesis that the four square step test would uniquely explain the between-participant variance in measures of mobility above and beyond that observed with muscle strength and mass was supported. These findings underscore the multifactorial role of lower extremity motor function as an important factor in mobility function in older adults, though the degree of unexplained variance indicates that further work is needed to identify new factors.

## Supplementary information

**Additional file 1: Table 1.** Inclusion/Exclusion criteria for older adults.

**Additional file 2: Table 2.** Overall model of best fit selection.

**Additional file 3: Table 3.** Model of best fit selection for sex (males).

**Additional file 4: Table 4.** Model of best fit selection for sex (females).

## Data Availability

All data generated or analyzed during this study are available from the corresponding author on reasonable request.

## Abbreviations

AIC: Akaike’s Information Criterion
App: Appendicular
BIA: Bioelectrical Impedance Analysis
BMI: Body Mass Index
BW: Body Weight
CFT: Complex Functional Task
CT: Computerized Tomography
Dep: Dependent
Dist: Distance
D-W: Durbin-Watson
Ht: Height
Isokin: Isokinetic
Isomet: Isometric
M: Meters
Min: Minute
MRI: Magnetic Resonance Imaging
MPC: Mallows’ Prediction Criterion
MVC: Maximum Voluntary Contraction
N: Newton
Pred: Predictor
RBANS: Repeatable Battery for the Assessment of Neuropsychological Status
SBC: Schwarz Bayesian Criterion
Sec: Second
S-P: Semi-partial
SPPB: Short Physical Performance Battery
Sq: Square
SPSS: Statistical Package for Social Sciences
Var: Variable
VIF: Variance Inflation Factor

## References

[1] Studenski S, Perera S, Patel K (2011). Gait speed and survival in older adults. JAMA..

[2] Cummings S, Studenski S, Ferrucci L (2014). A diagnosis of dismobility--giving mobility clinical visibility: a mobility working group recommendation. JAMA..

[3] Gill T, Allore H, Hardy S, Guo Z (2006). The dynamic nature of mobility disability in older persons. J Am Geriatr Soc.

[4] Newman A, Simonsick E, Naydeck B (2006). Association of long-distance corridor walk performance with mortality, cardiovascular disease, mobility limitation, and disability. JAMA..

[5] Simonsick E, Newman A, Visser M (2008). Mobility limitation in self-described well-functioning older adults: importance of endurance walk testing. J Gerontol a biol Sci med. Sci..

[6] Cawthon P, Fox K, Gandra S (2009). Do muscle mass, muscle density, strength, and physical function similarly influence risk of hospitalization in older adults?. J Am Geriatr Soc.

[7] Vermeulen J, Neyens J, van Rossum E (2011). Spreeuwenberg, M, de Witte L. Predicting ADL disability in community-dwelling elderly people using physical frailty indicators: a systematic review BMC Geriatrics.

[8] Hardy S, Kang Y, Studenski S, Degenholtz H (2011). Ability to walk 1/4 mile predicts subsequent disability, mortality, and health care costs. J Gen Intern Med.

[9] Shumway-Cook A, Ciol M, Yorkston K, Hoffman J, Chan L (2005). Mobility limitations in the Medicare population: prevalence and sociodemographic and clinical correlates. J Am Geriatr Soc.

[10] Musich S, Wang S, Ruiz J, Hawkins K, Wicker E (2018). The impact of mobility limitations on health outcomes among older adults. Geriatr Nurs.

[11] Rosenberg IH (1997). Sarcopenia: origins and clinical relevance. J Nutrition.

[12] Evans W. What is sarcopenia? J. Gerontol A Biol Sci Med Sci. 1995; 50 Spec No: 5–8. 10.1093/gerona/50a.special_issue.5 7493218

[13] Cawthon P, Travison T, Manini T, et al. Establishing the link between lean mass and grip strength cut-points with mobility disability and other health outcomes: proceedings of the sarcopenia definitions and outcomes consortium conference. J Gerontol A Biol Sci Med Sci. 209b; 10.1093/gerona/glz081. 10.1093/gerona/glz081 PMC7447857 30869772

[14] Motor Function – Interactive Guide to Physical Therapist Practice 3.0. (2014). American Physical Therapy Association. http://guidetoptpractice.apta.org/content/1/ SEC19.extract*.*.

[15] Dite W, Temple V (2002). A clinical test of stepping and change of direction to identify multiply falling older adults. Arch Phys Med Rehabil.

[16] Moore M, Barker K (2017). The validity and reliability of the four square step test in different adult populations: a systematic review. Syst Rev.

[17] Jung H, Yamasaki M (2016). Association of lower extremity range of motion and muscle strength with physical performance of community-dwelling older women. J Physiol Anthropol.

[18] Saito A, Wakasa M, Kimoto M (2019). Age-related changes in muscle elasticity and thickness of the lower extremities are associated with physical functions among community-dwelling older women. Geriatr Gerontol Int.

[19] Guralnik J, Simonsick E, Ferrucci L (1994). A short physical performance battery assessing lower extremity function: association with self-reported disability and prediction of mortality and nursing home admission. J Gerontol.

[20] Gómez J, Curcio C, Alvarado B, Zunzunegui M, Guralnik J (2013). Validity and reliability of the short physical performance battery (SPPB): a pilot study on mobility in the Colombian Andes. Colomb Med (Cali).

[21] Corbett D, Valiani V, Knaggs J, Manini T (2016). Evaluating walking intensity with hip-worn accelerometers in elders. Med Sci Sports Exerc.

[22] Randolph C, Tierney M, Mohr E, Chase T (1998). The repeatable battery for the assessment of neuropsychological status (RBANS): preliminary clinical validity. J Clin Exp Neuropsychol.

[23] Charlson M, Pompei P, Ales K, MacKenzie C (1987). A new method of classifying prognostic comorbidity in longitudinal studies: development and validation. J Chronic Dis.

[24] Tavoian D, Ampomah K, Amano S, Law T, Clark B (2019). Changes in DXA-derived lean mass and MRI-derived cross-sectional area of the thigh are modestly associated. Sci Rep.

[25] Hangartner T, Warner S, Braillon P, Jankowski L, Shepard J (2013). The official positions of the international society for clinical densitometry: acquisition of dual-energy X-ray absorptiometry body composition and considerations regarding analysis and repeatability of measures. J Clin Densitom.

[26] Manini T, Visser M, Won-Park S (2007). Knee extension strength cutpoints for maintaining mobility. J Am Geriatr Soc.

[27] Villafañe J, Valdes K, Vanti C, Pillastrini P, Borboni A. Reliability of handgrip strength test in elderly subjects with unilateral thumb carpometacarpal osteoarthritis. Hand (N Y). 215; 10(2): 205–209. 10.1007/s11552-014-9678-y PMC4447675 26034431

[28] O’brien R (2007). A caution regarding rules of thumb for variance inflation factors. Qual Quant.

[29] Zientek L, Thompson B (2006). Commonality analysis: partitioning variance to facilitate better understanding of data. JEI..

[30] Schwarz G (1978). Estimating the dimension of a model. Ann Stat.

[31] Akaike H (1987). Factor analysis and AIC. Psychometrika..

[32] Schafer J (1997). Analysis of incomplete multivariate data.

[33] Little R, Rubin B (2002). Statistical analysis with missing data 2nd edition.

[34] Faul F, Erdfelder E, Buchner A, Land A-G (2009). Statistical power analyses using G*power 3.1: tests for correlation and regression analyses. Behav Res Methods.

[35] Janssen I, Heymsfield S, Ross R. Low relative skeletal muscle mass (sarcopenia) in older persons is associated with functional impairment and physical disability. J Am Geriatr Soc. 2002; (50)5: 889–896. 10.1046/j.1532-5415.2002.50216.x 12028177

[36] Visser M, Goodpaster B, Kritchevsky S (2005). Muscle mass, muscle strength, and muscle fat infiltration as predictors of incident mobility limitations in well-functioning older persons. J Gerontol Series A.

[37] Reid K, Naumova E, Carabello R, Philips E, Fielding R (2008). Lower extremity muscle mass predicts functional performance in mobility-limited elders. J Nutr Health Aging.

[38] Curcio F, Basile C, Liguori I, Della-Morte D, Gargiulo G. Tinetti mobility test is related to muscle mass ad strength in non-institutionalized elderly people. Age (Dordr). 2016; 38: 525–533. 10.1007/s11357-016-9935-9 PMC5266213 27566307

[39] Schaap L, Koster A, Visser M (2013). Adiposity, muscle mass, and muscle strength in relation to functional decline in older persons. Epidemiol Rev.

[40] Cawthon P, Orwoll E, Peters K (2019). Strong relation between muscle mass determined by D3-creatine dilution, physical performance and incidence of falls and mobility limitations in a prospective cohort of older men. J Gerontol A Biol Sci Med Sci.

[41] Stoessl A, Lehericy S, Strafella A (2014). Imaging insights into basal ganglia function, Parkinson’s disease, and dystonia. Lancet..

[42] Mogenson G, Jones D, Yim C (1980). From motivation to action: functional interface between the limbic system and the motor system. Prog Neurobiol.

[43] Lord S, Fitzpatrick R (2001). Choice stepping reaction time: a composite measure of falls risk in older people. J Gerontol A Biol Sci Med Sci.

[44] Pijnappels M, Delbaere K, Sturnieks D, Lord S (2010). The association between choice stepping reaction time and falls in older adults—a path analysis model. Age Aging.

[45] Duncan R, Earhart G. Four square step test performance in people with Parkinson’s disease. Physical Therapy Faculty Publication. 2013; Paper 31: http://digitalcommons.wustl.edu/ pt_facpubs/3. 10.1097/NPT.0b013e31827f0d7a 23364168

[46] Wagner J, Norris R, Van Dillen L, Thomas F, Naismith R (2013). Four square step test in ambulant persons with multiple sclerosis: validity, reliability, and responsiveness. Int J Rehabil Res.

[47] Pool D, Valentine J, Bear N, Donnelly C, Elliott C, Stannage K (2015). The orthotic and therapeutic effects following daily community applied functional electrical stimulation in children with unilateral spastic cerebral palsy: a randomized controlled trial. BMC Pediatr.

[48] Clark B, Tavoian D, Goodpaster B, Cawthon P, Hensen R, Manini T (2018). Comment on “pitfalls in the measurement of muscle mass: a need for a reference standard” by Buckinx et al. J Cachexia Sarcopenia Muscle.

[49] Evans W, Hellerstein M, Orwoll E, Cummings S, Cawthon P (2019). D_3_ -creatine dilution and the importance of accuracy in the assessment of skeletal muscle mass. J Cachexia Sarcopenia Muscle.

[50] Wang W, Wang Z, Faith M, Kotler D, Shih R, Heysmfield S (1999). Regional skeletal muscle measurement: evaluation of new dual-energy X-ray absorptiometry model. J Appl Physiol.

[51] Hanson R, Williamson D, Finnegan T (2007). Estimation of thigh cross-sectional area by dual-energy X-ray absorptiometry in frail elderly patients. Am J Clin Nutr.

[52] Freda P, Shen W, Reyes-Vidal C (2009). Skeletal muscle mass in acromegaly assessed by magnetic resonance imaging and dual-photon x-ray absorptiometry. J Clin Endocrinol Metab.

