# Supplementary material for: Relative contribution of muscle strength, lean mass, and lower extremity motor function in explaining between-person variance in mobility in older adults
Source: BMC Geriatr. 2020 Jul 28;20:255. doi: 10.1186/s12877-020-01656-y (PMC7385889; doi:10.1186/s12877-020-01656-y)
Supplement: Supplementary file 1 — Additional file 1: Table 1. Inclusion/Exclusion criteria for older adults. [file 12877_2020_1656_MOESM1_ESM.docx]

| **INCLUSION** |
| --- |
| Age 60+ years (older adults) with no significant health issues or conditions that, in the investigator's opinion, would limit the subject's ability to complete the study per protocol or that would impact the capability to get an accurate measurement of study endpoints. |
| Body mass index between 18 and 40 kg/m^2^. |
| Willingness to undergo all testing procedures. |
| Able to read, understand, and complete study-related questionnaires. |
| Able to read and understand, and willing to sign the informed consent form (ICF). |
|  |
| **EXCLUSION** |
| Failure to provide informed consent. |
| Known neuromuscular or neurological conditions affecting somatosensory or motor function or control (e.g., hemiplegia, multiple sclerosis, peripheral neuropathy, Parkinson’s disease, Myasthenia Gravis, Ataxia, Apraxia, mitochondrial myopathy, etc.). |
| Unable to communicate because of severe hearing loss or speech disorder. |
| Severe visual impairment, which would preclude completion of the assessments. |
| Cancer requiring treatment currently or in the past 2 years (except primary non-melanoma skin cancer or in situ cervical cancer). |
| Any ADL disability. |
| Recent unexplained weight loss (> 10 pounds in past month). |
| Hospitalization (medical confinement for 24 hours), or immobilization, or major surgical procedure requiring general anesthesia within 12 weeks prior to screening, or any planned surgical procedures during the study period. |
| Chronic or relapsing/remitting gastrointestinal disorders such as inflammatory bowel disease and irritable bowel syndrome. |
| Known history of human immunodeficiency virus (HIV) antibody at screening. |
| Use of systemic glucocorticoids. |
| Severe pulmonary disease, requiring either steroid pills or injections or the use of  supplemental oxygen. |
| Severe cardiac disease, including NYHA Class III or IV congestive heart failure, clinically significant aortic stenosis, recent history of cardiac arrest (within 6-months), use of a cardiac defibrillator, or uncontrolled angina. |
| Renal failure on hemodialysis. |
| Psychiatric conditions that warrant acute or chronic therapeutic intervention (e.g., major depressive disorder, bipolar disorder, panic disorder, schizophrenia) that in the investigator’s opinion interfered with the conduct of study procedures. |
| Unable to undergo Magnetic Resonance Imaging (MRI), Transcranial Magnetic Stimulation (TMS), or DEXA (e. g. body containing any metallic medical devices or equipment, including heart pacemakers, metal prostheses, implants or surgical clips, any prior injury from shrapnel or grinding metal, exposure to metallic dusts, metallic shavings or having tattoos containing metallic dyes, body dimensions exceeding capacity of MRI or DEXA). Note: This manuscript is an analysis from a larger study and the MRI and brain stimulation exclusion criteria, which are not presented here, were part of this larger study. |
| Unable to reliably undergo exercise or strength tests described for this study. |
| Participation in any clinical trial within 12 weeks prior to screening. |
| Limb amputation (except for toes) and/or any fracture within 24 weeks of study screening. |
| Conditions (such as myasthenia gravis, myositis, muscular dystrophy or myopathy, including drug-induced myopathy) leading to muscle loss, muscle weakness, muscle cramps or myalgia. |
| Acute viral or bacterial upper or lower respiratory infection at screening. |
| Abnormal or uncontrolled blood pressure at the screening visit defined as BP > 170/100 mmHg. If taking anti-hypertensive medication, had to have been on stable doses of medication for more than 3 months. |
| Medications known to alter TMS-based outcomes (Ziemann, Clin Neurophysiol, 115: 1717-1729, 2004). For instance, individuals taking benzodiazipines were excluded from study participation. Note: This exclusion criteria was related to the larger study. |
| Current or recent history (within 1 year of screen) of heavy alcohol consumption or drug abuse that in the investigator’s opinion interfered with the conduct of study procedures. |
| Self-reported engagement in structured aerobic physical activity > 360 minutes/week. |
| Self-reported engagement in progressive resistance exercise training (defined as progressive resistance exercise over the past 3-months at a frequency greater than or equal to 4 days per week. |
