# Supplementary material for: Relative contribution of muscle strength, lean mass, and lower extremity motor function in explaining between-person variance in mobility in older adults
Source: BMC Geriatr. 2020 Jul 28;20:255. doi: 10.1186/s12877-020-01656-y (PMC7385889; doi:10.1186/s12877-020-01656-y)
Supplement: Supplementary file 2 — Additional file 2: Table 2. Overall model of best fit selection. [file 12877_2020_1656_MOESM2_ESM.docx]

| **DEPENDENT VARIBLE – 6-MIN WALK GAIT SPEED** | | | | | |
| --- | --- | --- | --- | --- | --- |
| **INDEPENDENT VARIABLES** | | | **SELECTION CRITERIA** | | |
| **Muscle Mass** | **Muscle Strength** | **Motor Function** | **AIC** | **MPC** | **SBC** |
| Lower Limb Muscle Mass | Isokinetic Strength / BW | Four Sq. Step Test | 681.255 | 4.000 | 690.833 |
| Total Muscle Mass | Isokinetic Strength / BW | Four Sq. Step Test | 681.652 | 4.000 | 691.230 |
| Appendicular Lean Mass | Isokinetic Strength / BW | Four Sq. Step Test | 673.302 | 4.000 | 682.830 |
| Appendicular Lean Mass / Ht^2^ | Isokinetic Strength / BW | Four Sq. Step Test | 681.618 | 4.000 | 691.195 |
| Appendicular Lean Mass / BW | Isokinetic Strength / BW | Four Sq. Step Test | 673.531 | 4.000 | 683.059 |
| Appendicular Lean Mass / BMI | Isokinetic Strength / BW | Four Sq. Step Test | 673.404 | 4.000 | 682.933 |
|  |  |  |  |  |  |
| Lower Limb Muscle Mass | Isokinetic Strength / BMI | Four Sq. Step Test | 687.479 | 4.000 | 697.057 |
| Total Muscle Mass | Isokinetic Strength / BMI | Four Sq. Step Test | 686.276 | 4.000 | 695.854 |
| Appendicular Lean Mass | Isokinetic Strength / BMI | Four Sq. Step Test | 679.134 | 4.000 | 688.662 |
| Appendicular Lean Mass / Ht^2^ | Isokinetic Strength / BMI | Four Sq. Step Test | 687.553 | 4.000 | 697.131 |
| Appendicular Lean Mass / BW | Isokinetic Strength / BMI | Four Sq. Step Test | 678.789 | 4.000 | 688.317 |
| Appendicular Lean Mass / BMI | Isokinetic Strength / BMI | Four Sq. Step Test | 679.377 | 4.000 | 688.906 |
|  |  |  |  |  |  |
| Lower Limb Muscle Mass | Isokinetic Strength | Four Sq. Step Test | 694.515 | 4.000 | 704.093 |
| Total Muscle Mass | Isokinetic Strength | Four Sq. Step Test | 692.657 | 4.000 | 702.235 |
| Appendicular Lean Mass | Isokinetic Strength | Four Sq. Step Test | 685.376 | 4.000 | 694.905 |
| Appendicular Lean Mass / Ht^2^ | Isokinetic Strength | Four Sq. Step Test | 693.902 | 4.000 | 703.480 |
| Appendicular Lean Mass / BW | Isokinetic Strength | Four Sq. Step Test | 682.228 | 4.000 | 691.756 |
| Appendicular Lean Mass / BMI | Isokinetic Strength | Four Sq. Step Test | 685.919 | 4.000 | 695.447 |
|  |  |  |  |  |  |
| Lower Limb Muscle Mass | Isometric Strength / BW | Four Sq. Step Test | 699.432 | 4.000 | 709.010 |
| Total Muscle Mass | Isometric Strength / BW | Four Sq. Step Test | 699.979 | 4.000 | 709.556 |
| Appendicular Lean Mass | Isometric Strength / BW | Four Sq. Step Test | 690.629 | 4.000 | 700.157 |
| Appendicular Lean Mass / Ht^2^ | Isometric Strength / BW | Four Sq. Step Test | 699.963 | 4.000 | 709.541 |
| Appendicular Lean Mass / BW | Isometric Strength / BW | Four Sq. Step Test | 686.643 | 4.000 | 696.171 |
| Appendicular Lean Mass / BMI | Isometric Strength / BW | Four Sq. Step Test | 687.700 | 4.000 | 697.228 |
|  |  |  |  |  |  |
| Lower Limb Muscle Mass | Isometric Strength / BMI | Four Sq. Step Test | 700.598 | 4.000 | 710.176 |
| Total Muscle Mass | Isometric Strength / BMI | Four Sq. Step Test | 700.025 | 4.000 | 709.603 |
| Appendicular Lean Mass | Isometric Strength / BMI | Four Sq. Step Test | 691.808 | 4.000 | 701.336 |
| Appendicular Lean Mass / Ht^2^ | Isometric Strength / BMI | Four Sq. Step Test | 700.575 | 4.000 | 710.153 |
| Appendicular Lean Mass / BW | Isometric Strength / BMI | Four Sq. Step Test | 686.870 | 4.000 | 696.398 |
| Appendicular Lean Mass / BMI | Isometric Strength / BMI | Four Sq. Step Test | 689.010 | 4.000 | 698.538 |

| Lower Limb Muscle Mass | Isometric Strength | Four Sq. Step Test | 705.552 | 4.000 | 715.129 |
| --- | --- | --- | --- | --- | --- |
| Total Muscle Mass | Isometric Strength | Four Sq. Step Test | 704.746 | 4.000 | 714.324 |
| Appendicular Lean Mass | Isometric Strength | Four Sq. Step Test | 696.071 | 4.000 | 705.599 |
| Appendicular Lean Mass / Ht^2^ | Isometric Strength | Four Sq. Step Test | 705.218 | 4.000 | 714.796 |
| Appendicular Lean Mass / BW | Isometric Strength | Four Sq. Step Test | 687.245 | 4.000 | 696.773 |
| Appendicular Lean Mass / BMI | Isometric Strength | Four Sq. Step Test | 689.272 | 4.000 | 698.800 |
|  |  |  |  |  |  |
| Lower Limb Muscle Mass | Handgrip Strength / BW | Four Sq. Step Test | 703.289 | 4.000 | 712.916 |
| Total Muscle Mass | Handgrip Strength / BW | Four Sq. Step Test | 704.100 | 4.000 | 713.727 |
| Appendicular Lean Mass | Handgrip Strength / BW | Four Sq. Step Test | 694.553 | 4.000 | 704.130 |
| Appendicular Lean Mass / Ht^2^ | Handgrip Strength / BW | Four Sq. Step Test | 704.023 | 4.000 | 713.650 |
| Appendicular Lean Mass / BW | Handgrip Strength / BW | Four Sq. Step Test | 692.275 | 4.000 | 701.853 |
| Appendicular Lean Mass / BMI | Handgrip Strength / BW | Four Sq. Step Test | 692.624 | 4.000 | 702.202 |
|  |  |  |  |  |  |
| Lower Limb Muscle Mass | Handgrip Strength / BMI | Four Sq. Step Test | 706.501 | 4.000 | 716.128 |
| Total Muscle Mass | Handgrip Strength / BMI | Four Sq. Step Test | 706.145 | 4.000 | 715.772 |
| Appendicular Lean Mass | Handgrip Strength / BMI | Four Sq. Step Test | 697.656 | 4.000 | 707.234 |
| Appendicular Lean Mass / Ht^2^ | Handgrip Strength / BMI | Four Sq. Step Test | 706.550 | 4.000 | 716.131 |
| Appendicular Lean Mass / BW | Handgrip Strength / BMI | Four Sq. Step Test | 693.600 | 4.000 | 703.178 |
| Appendicular Lean Mass / BMI | Handgrip Strength / BMI | Four Sq. Step Test | 695.771 | 4.000 | 705.349 |
|  |  |  |  |  |  |
| Lower Limb Muscle Mass | Handgrip Strength | Four Sq. Step Test | 711.939 | 4.000 | 721.566 |
| Total Muscle Mass | Handgrip Strength | Four Sq. Step Test | 710.745 | 4.000 | 720.372 |
| Appendicular Lean Mass | Handgrip Strength | Four Sq. Step Test | 702.243 | 4.000 | 711.821 |
| Appendicular Lean Mass / Ht^2^ | Handgrip Strength | Four Sq. Step Test | 711.113 | 4.000 | 720.740 |
| Appendicular Lean Mass / BW | Handgrip Strength | Four Sq. Step Test | 693.878 | 4.000 | 703.455 |
| Appendicular Lean Mass / BMI | Handgrip Strength | Four Sq. Step Test | 696.954 | 4.000 | 706.532 |
|  |  |  |  |  |  |
| **DEPENDENT VARIBLE – STAIR CLIMB POWER** | | | | | |
| Lower Limb Muscle Mass | Isokinetic Strength / BW | Four Sq. Step Test | 557.539 | 4.000 | 567.117 |
| Total Muscle Mass | Isokinetic Strength / BW | Four Sq. Step Test | 557.290 | 4.000 | 579.868 |
| Appendicular Lean Mass | Isokinetic Strength / BW | Four Sq. Step Test | 552.259 | 4.000 | 561.787 |
| Appendicular Lean Mass / Ht^2^ | Isokinetic Strength / BW | Four Sq. Step Test | 563.112 | 4.000 | 572.690 |
| Appendicular Lean Mass / BW | Isokinetic Strength / BW | Four Sq. Step Test | 559.800 | 4.000 | 609.328 |
| Appendicular Lean Mass / BMI | Isokinetic Strength / BW | Four Sq. Step Test | 592.773 | 4.000 | 602.301 |

| Lower Limb Muscle Mass | Isokinetic Strength / BMI | Four Sq. Step Test | 557.684 | 4.000 | 567.262 |
| --- | --- | --- | --- | --- | --- |
| Total Muscle Mass | Isokinetic Strength / BMI | Four Sq. Step Test | 570.464 | 4.000 | 580.042 |
| Appendicular Lean Mass | Isokinetic Strength / BMI | Four Sq. Step Test | 551.924 | 4.000 | 561.452 |
| Appendicular Lean Mass / Ht^2^ | Isokinetic Strength / BMI | Four Sq. Step Test | 562.077 | 4.000 | 571.655 |
| Appendicular Lean Mass / BW | Isokinetic Strength / BMI | Four Sq. Step Test | 594.361 | 4.000 | 603.889 |
| Appendicular Lean Mass / BMI | Isokinetic Strength / BMI | Four Sq. Step Test | 592.882 | 4.000 | 602.410 |
| Lower Limb Muscle Mass | Isokinetic Strength / BMI | Four Sq. Step Test | 557.684 | 4.000 | 567.262 |
|  |  |  |  |  |  |
| Lower Limb Muscle Mass | Isokinetic Strength | Four Sq. Step Test | 554.217 | 4.000 | 563.795 |
| Total Muscle Mass | Isokinetic Strength | Four Sq. Step Test | 562.746 | 4.000 | 572.324 |
| Appendicular Lean Mass | Isokinetic Strength | Four Sq. Step Test | 549.752 | 4.000 | 559.280 |
| Appendicular Lean Mass / Ht^2^ | Isokinetic Strength | Four Sq. Step Test | 553.728 | 4.000 | 563.306 |
| Appendicular Lean Mass / BW | Isokinetic Strength | Four Sq. Step Test | 564.444 | 4.000 | 573.972 |
| Appendicular Lean Mass / BMI | Isokinetic Strength | Four Sq. Step Test | 567.019 | 4.000 | 576.548 |
|  |  |  |  |  |  |
| Lower Limb Muscle Mass | Isometric Strength / BW | Four Sq. Step Test | 551.223 | 4.000 | 560.801 |
| Total Muscle Mass | Isometric Strength / BW | Four Sq. Step Test | 566.879 | 4.000 | 576.457 |
| Appendicular Lean Mass | Isometric Strength / BW | Four Sq. Step Test | 546.131 | 4.000 | 555.659 |
| Appendicular Lean Mass / Ht^2^ | Isometric Strength / BW | Four Sq. Step Test | 561.698 | 4.000 | 571.275 |
| Appendicular Lean Mass / BW | Isometric Strength / BW | Four Sq. Step Test | 601.124 | 4.000 | 610.652 |
| Appendicular Lean Mass / BMI | Isometric Strength / BW | Four Sq. Step Test | 591.995 | 4.000 | 601.523 |
|  |  |  |  |  |  |
| Lower Limb Muscle Mass | Isometric Strength / BMI | Four Sq. Step Test | 550.379 | 4.000 | 559.957 |
| Total Muscle Mass | Isometric Strength / BMI | Four Sq. Step Test | 566.901 | 4.000 | 576.478 |
| Appendicular Lean Mass | Isometric Strength / BMI | Four Sq. Step Test | 544.704 | 4.000 | 554.232 |
| Appendicular Lean Mass / Ht^2^ | Isometric Strength / BMI | Four Sq. Step Test | 561.491 | 4.000 | 571.068 |
| Appendicular Lean Mass / BW | Isometric Strength / BMI | Four Sq. Step Test | 597.720 | 4.000 | 607.248 |
| Appendicular Lean Mass / BMI | Isometric Strength / BMI | Four Sq. Step Test | 593.204 | 4.000 | 602.733 |
|  |  |  |  |  |  |
| Lower Limb Muscle Mass | Isometric Strength | Four Sq. Step Test | 552.038 | 4.000 | 561.616 |
| Total Muscle Mass | Isometric Strength | Four Sq. Step Test | 568.298 | 4.000 | 577.876 |
| Appendicular Lean Mass | Isometric Strength | Four Sq. Step Test | 547.682 | 4.000 | 557.210 |
| Appendicular Lean Mass / Ht^2^ | Isometric Strength | Four Sq. Step Test | 558.794 | 4.000 | 568.372 |
| Appendicular Lean Mass / BW | Isometric Strength | Four Sq. Step Test | 578.659 | 4.000 | 588.188 |
| Appendicular Lean Mass / BMI | Isometric Strength | Four Sq. Step Test | 581.102 | 4.000 | 590.630 |

| Lower Limb Muscle Mass | Handgrip Strength / BW | Four Sq. Step Test | 564.476 | 4.000 | 574.103 |
| --- | --- | --- | --- | --- | --- |
| Total Muscle Mass | Handgrip Strength / BW | Four Sq. Step Test | 577.793 | 4.000 | 587.420 |
| Appendicular Lean Mass | Handgrip Strength / BW | Four Sq. Step Test | 558.353 | 4.000 | 567.930 |
| Appendicular Lean Mass / Ht^2^ | Handgrip Strength / BW | Four Sq. Step Test | 573.620 | 4.000 | 583.247 |
| Appendicular Lean Mass / BW | Handgrip Strength / BW | Four Sq. Step Test | 608.471 | 4.000 | 618.049 |
| Appendicular Lean Mass / BMI | Handgrip Strength / BW | Four Sq. Step Test | 597.764 | 4.000 | 607.341 |
|  |  |  |  |  |  |
| Lower Limb Muscle Mass | Handgrip Strength / BMI | Four Sq. Step Test | 563.710 | 4.000 | 573.337 |
| Total Muscle Mass | Handgrip Strength / BMI | Four Sq. Step Test | 577.403 | 4.000 | 587.030 |
| Appendicular Lean Mass | Handgrip Strength / BMI | Four Sq. Step Test | 556.787 | 4.000 | 566.365 |
| Appendicular Lean Mass / Ht^2^ | Handgrip Strength / BMI | Four Sq. Step Test | 573.428 | 4.000 | 583.055 |
| Appendicular Lean Mass / BW | Handgrip Strength / BMI | Four Sq. Step Test | 607.615 | 4.000 | 617.193 |
| Appendicular Lean Mass / BMI | Handgrip Strength / BMI | Four Sq. Step Test | 599.220 | 4.000 | 608.797 |
|  |  |  |  |  |  |
| Lower Limb Muscle Mass | Handgrip Strength | Four Sq. Step Test | 564.376 | 4.000 | 574.003 |
| Total Muscle Mass | Handgrip Strength | Four Sq. Step Test | 576.354 | 4.000 | 585.981 |
| Appendicular Lean Mass | Handgrip Strength | Four Sq. Step Test | 559.429 | 4.000 | 569.007 |
| Appendicular Lean Mass / Ht^2^ | Handgrip Strength | Four Sq. Step Test | 569.197 | 4.000 | 578.824 |
| Appendicular Lean Mass / BW | Handgrip Strength | Four Sq. Step Test | 584.455 | 4.000 | 594.023 |
| Appendicular Lean Mass / BMI | Handgrip Strength | Four Sq. Step Test | 585.996 | 4.000 | 595.574 |
|  |  |  |  |  |  |
| **DEPENDENT VARIBLE – 5x CHAIR RISE TIME** | |  | | | |
| Lower Limb Muscle Mass | Isokinetic Strength / BW | Four Sq. Step Test | 196.784 | 4.000 | 206.362 |
| Total Muscle Mass | Isokinetic Strength / BW | Four Sq. Step Test | 197.061 | 4.000 | 206.639 |
| Appendicular Lean Mass | Isokinetic Strength / BW | Four Sq. Step Test | 195.154 | 4.000 | 204.682 |
| Appendicular Lean Mass / Ht^2^ | Isokinetic Strength / BW | Four Sq. Step Test | 197.398 | 4.000 | 206.976 |
| Appendicular Lean Mass / BW | Isokinetic Strength / BW | Four Sq. Step Test | 195.698 | 4.000 | 205.226 |
| Appendicular Lean Mass / BMI | Isokinetic Strength / BW | Four Sq. Step Test | 193.641 | 4.000 | 203.169 |
|  |  |  |  |  |  |
| Lower Limb Muscle Mass | Isokinetic Strength / BMI | Four Sq. Step Test | 200.327 | 4.000 | 209.905 |
| Total Muscle Mass | Isokinetic Strength / BMI | Four Sq. Step Test | 200.835 | 4.000 | 210.413 |
| Appendicular Lean Mass | Isokinetic Strength / BMI | Four Sq. Step Test | 198.480 | 4.000 | 208.008 |
| Appendicular Lean Mass / Ht^2^ | Isokinetic Strength / BMI | Four Sq. Step Test | 201.776 | 4.000 | 211.353 |
| Appendicular Lean Mass / BW | Isokinetic Strength / BMI | Four Sq. Step Test | 200.482 | 4.000 | 210.010 |
| Appendicular Lean Mass / BMI | Isokinetic Strength / BMI | Four Sq. Step Test | 197.242 | 4.000 | 206.770 |

| Lower Limb Muscle Mass | Isokinetic Strength | Four Sq. Step Test | 199.249 | 4.000 | 208.827 |
| --- | --- | --- | --- | --- | --- |
| Total Muscle Mass | Isokinetic Strength | Four Sq. Step Test | 200.921 | 4.000 | 210.499 |
| Appendicular Lean Mass | Isokinetic Strength | Four Sq. Step Test | 196.984 | 4.000 | 206.512 |
| Appendicular Lean Mass / Ht^2^ | Isokinetic Strength | Four Sq. Step Test | 202.290 | 4.000 | 211.867 |
| Appendicular Lean Mass / BW | Isokinetic Strength | Four Sq. Step Test | 201.239 | 4.000 | 210.767 |
| Appendicular Lean Mass / BMI | Isokinetic Strength | Four Sq. Step Test | 201.210 | 4.000 | 210.738 |
|  |  |  |  |  |  |
| Lower Limb Muscle Mass | Isometric Strength / BW | Four Sq. Step Test | 196.720 | 4.000 | 206.298 |
| Total Muscle Mass | Isometric Strength / BW | Four Sq. Step Test | 196.465 | 4.000 | 206.042 |
| Appendicular Lean Mass | Isometric Strength / BW | Four Sq. Step Test | 195.080 | 4.000 | 204.608 |
| Appendicular Lean Mass / Ht^2^ | Isometric Strength / BW | Four Sq. Step Test | 197.441 | 4.000 | 207.019 |
| Appendicular Lean Mass / BW | Isometric Strength / BW | Four Sq. Step Test | 196.040 | 4.000 | 205.568 |
| Appendicular Lean Mass / BMI | Isometric Strength / BW | Four Sq. Step Test | 194.117 | 4.000 | 203.645 |
|  |  |  |  |  |  |
| Lower Limb Muscle Mass | Isometric Strength / BMI | Four Sq. Step Test | 199.521 | 4.000 | 209.099 |
| Total Muscle Mass | Isometric Strength / BMI | Four Sq. Step Test | 199.435 | 4.000 | 209.013 |
| Appendicular Lean Mass | Isometric Strength / BMI | Four Sq. Step Test | 197.673 | 4.000 | 207.201 |
| Appendicular Lean Mass / Ht^2^ | Isometric Strength / BMI | Four Sq. Step Test | 201.098 | 4.000 | 210.676 |
| Appendicular Lean Mass / BW | Isometric Strength / BMI | Four Sq. Step Test | 200.036 | 4.000 | 209.564 |
| Appendicular Lean Mass / BMI | Isometric Strength / BMI | Four Sq. Step Test | 196.900 | 4.000 | 206.428 |
|  |  |  |  |  |  |
| Lower Limb Muscle Mass | Isometric Strength | Four Sq. Step Test | 198.279 | 4.000 | 207.857 |
| Total Muscle Mass | Isometric Strength | Four Sq. Step Test | 198.166 | 4.000 | 207.744 |
| Appendicular Lean Mass | Isometric Strength | Four Sq. Step Test | 196.144 | 4.000 | 205.672 |
| Appendicular Lean Mass / Ht^2^ | Isometric Strength | Four Sq. Step Test | 201.269 | 4.000 | 210.847 |
| Appendicular Lean Mass / BW | Isometric Strength | Four Sq. Step Test | 200.821 | 4.000 | 210.349 |
| Appendicular Lean Mass / BMI | Isometric Strength | Four Sq. Step Test | 200.378 | 4.000 | 209.906 |
|  |  |  |  |  |  |
| Lower Limb Muscle Mass | Handgrip Strength / BW | Four Sq. Step Test | 200.421 | 4.000 | 210.047 |
| Total Muscle Mass | Handgrip Strength / BW | Four Sq. Step Test | 200.467 | 4.000 | 210.093 |
| Appendicular Lean Mass | Handgrip Strength / BW | Four Sq. Step Test | 198.786 | 4.000 | 208.364 |
| Appendicular Lean Mass / Ht^2^ | Handgrip Strength / BW | Four Sq. Step Test | 200.765 | 4.000 | 210.392 |
| Appendicular Lean Mass / BW | Handgrip Strength / BW | Four Sq. Step Test | 199.254 | 4.000 | 208.832 |
| Appendicular Lean Mass / BMI | Handgrip Strength / BW | Four Sq. Step Test | 198.125 | 4.000 | 207.702 |

| Lower Limb Muscle Mass | Handgrip Strength / BMI | Four Sq. Step Test | 203.580 | 4.000 | 213.207 |
| --- | --- | --- | --- | --- | --- |
| Total Muscle Mass | Handgrip Strength / BMI | Four Sq. Step Test | 203.718 | 4.000 | 213.345 |
| Appendicular Lean Mass | Handgrip Strength / BMI | Four Sq. Step Test | 201.765 | 4.000 | 211.343 |
| Appendicular Lean Mass / Ht^2^ | Handgrip Strength / BMI | Four Sq. Step Test | 204.466 | 4.000 | 214.093 |
| Appendicular Lean Mass / BW | Handgrip Strength / BMI | Four Sq. Step Test | 202.866 | 4.000 | 212.444 |
| Appendicular Lean Mass / BMI | Handgrip Strength / BMI | Four Sq. Step Test | 201.483 | 4.000 | 211.061 |
|  |  |  |  |  |  |
| Lower Limb Muscle Mass | Handgrip Strength | Four Sq. Step Test | 202.891 | 4.000 | 212.518 |
| Total Muscle Mass | Handgrip Strength | Four Sq. Step Test | 203.666 | 4.000 | 213.292 |
| Appendicular Lean Mass | Handgrip Strength | Four Sq. Step Test | 200.557 | 4.000 | 210.135 |
| Appendicular Lean Mass / Ht^2^ | Handgrip Strength | Four Sq. Step Test | 204.910 | 4.000 | 214.536 |
| Appendicular Lean Mass / BW | Handgrip Strength | Four Sq. Step Test | 203.254 | 4.000 | 212.832 |
| Appendicular Lean Mass / BMI | Handgrip Strength | Four Sq. Step Test | 203.933 | 4.000 | 213.511 |
|  |  |  |  |  |  |
| **DEPENDENT VARIBLE – COMPLEX FUNCTION TEST** | |  | | | |
| Lower Limb Muscle Mass | Isokinetic Strength / BW | Four Sq. Step Test | 311.008 | 4.000 | 320.586 |
| Total Muscle Mass | Isokinetic Strength / BW | Four Sq. Step Test | 312.481 | 4.000 | 322.058 |
| Appendicular Lean Mass | Isokinetic Strength / BW | Four Sq. Step Test | 304.874 | 4.000 | 314.402 |
| Appendicular Lean Mass / Ht^2^ | Isokinetic Strength / BW | Four Sq. Step Test | 312.357 | 4.000 | 321.935 |
| Appendicular Lean Mass / BW | Isokinetic Strength / BW | Four Sq. Step Test | 308.458 | 4.000 | 317.986 |
| Appendicular Lean Mass / BMI | Isokinetic Strength / BW | Four Sq. Step Test | 309.146 | 4.000 | 318.674 |
|  |  |  |  |  |  |
| Lower Limb Muscle Mass | Isokinetic Strength / BMI | Four Sq. Step Test | 309.156 | 4.000 | 318.734 |
| Total Muscle Mass | Isokinetic Strength / BMI | Four Sq. Step Test | 311.410 | 4.000 | 320.988 |
| Appendicular Lean Mass | Isokinetic Strength / BMI | Four Sq. Step Test | 303.353 | 4.000 | 312.881 |
| Appendicular Lean Mass / Ht^2^ | Isokinetic Strength / BMI | Four Sq. Step Test | 313.401 | 4.000 | 322.979 |
| Appendicular Lean Mass / BW | Isokinetic Strength / BMI | Four Sq. Step Test | 310.076 | 4.000 | 319.604 |
| Appendicular Lean Mass / BMI | Isokinetic Strength / BMI | Four Sq. Step Test | 311.518 | 4.000 | 321.046 |
|  |  |  |  |  |  |
| Lower Limb Muscle Mass | Isokinetic Strength | Four Sq. Step Test | 315.371 | 4.000 | 324.949 |
| Total Muscle Mass | Isokinetic Strength | Four Sq. Step Test | 318.894 | 4.000 | 328.471 |
| Appendicular Lean Mass | Isokinetic Strength | Four Sq. Step Test | 309.300 | 4.000 | 318.828 |
| Appendicular Lean Mass / Ht^2^ | Isokinetic Strength | Four Sq. Step Test | 318.670 | 4.000 | 328.248 |
| Appendicular Lean Mass / BW | Isokinetic Strength | Four Sq. Step Test | 310.540 | 4.000 | 320.068 |
| Appendicular Lean Mass / BMI | Isokinetic Strength | Four Sq. Step Test | 313.435 | 4.000 | 322.963 |

| Lower Limb Muscle Mass | Isometric Strength / BW | Four Sq. Step Test | 312.797 | 4.000 | 322.375 |
| --- | --- | --- | --- | --- | --- |
| Total Muscle Mass | Isometric Strength / BW | Four Sq. Step Test | 312.696 | 4.000 | 322.273 |
| Appendicular Lean Mass | Isometric Strength / BW | Four Sq. Step Test | 305.556 | 4.000 | 315.084 |
| Appendicular Lean Mass / Ht^2^ | Isometric Strength / BW | Four Sq. Step Test | 313.386 | 4.000 | 322.964 |
| Appendicular Lean Mass / BW | Isometric Strength / BW | Four Sq. Step Test | 305.104 | 4.000 | 314.632 |
| Appendicular Lean Mass / BMI | Isometric Strength / BW | Four Sq. Step Test | 307.192 | 4.000 | 316.720 |
|  |  |  |  |  |  |
| Lower Limb Muscle Mass | Isometric Strength / BMI | Four Sq. Step Test | 311.882 | 4.000 | 321.46 |
| Total Muscle Mass | Isometric Strength / BMI | Four Sq. Step Test | 311.903 | 4.000 | 321.481 |
| Appendicular Lean Mass | Isometric Strength / BMI | Four Sq. Step Test | 304.973 | 4.000 | 314.501 |
| Appendicular Lean Mass / Ht^2^ | Isometric Strength / BMI | Four Sq. Step Test | 313.768 | 4.000 | 323.346 |
| Appendicular Lean Mass / BW | Isometric Strength / BMI | Four Sq. Step Test | 304.894 | 4.000 | 314.422 |
| Appendicular Lean Mass / BMI | Isometric Strength / BMI | Four Sq. Step Test | 307.562 | 4.000 | 317.090 |
|  |  |  |  |  |  |
| Lower Limb Muscle Mass | Isometric Strength | Four Sq. Step Test | 316.306 | 4.000 | 325.884 |
| Total Muscle Mass | Isometric Strength | Four Sq. Step Test | 316.870 | 4.000 | 326.448 |
| Appendicular Lean Mass | Isometric Strength | Four Sq. Step Test | 308.539 | 4.000 | 318.068 |
| Appendicular Lean Mass / Ht^2^ | Isometric Strength | Four Sq. Step Test | 316.900 | 4.000 | 326.478 |
| Appendicular Lean Mass / BW | Isometric Strength | Four Sq. Step Test | 302.124 | 4.000 | 311.653 |
| Appendicular Lean Mass / BMI | Isometric Strength | Four Sq. Step Test | 303.496 | 4.000 | 313.024 |
|  |  |  |  |  |  |
| Lower Limb Muscle Mass | Handgrip Strength / BW | Four Sq. Step Test | 318.219 | 4.000 | 327.846 |
| Total Muscle Mass | Handgrip Strength / BW | Four Sq. Step Test | 318.955 | 4.000 | 328.582 |
| Appendicular Lean Mass | Handgrip Strength / BW | Four Sq. Step Test | 311.357 | 4.000 | 320.934 |
| Appendicular Lean Mass / Ht^2^ | Handgrip Strength / BW | Four Sq. Step Test | 318.885 | 4.000 | 328.512 |
| Appendicular Lean Mass / BW | Handgrip Strength / BW | Four Sq. Step Test | 312.853 | 4.000 | 322.431 |
| Appendicular Lean Mass / BMI | Handgrip Strength / BW | Four Sq. Step Test | 314.282 | 4.000 | 323.860 |
|  |  |  |  |  |  |
| Lower Limb Muscle Mass | Handgrip Strength / BMI | Four Sq. Step Test | 317.474 | 4.000 | 327.101 |
| Total Muscle Mass | Handgrip Strength / BMI | Four Sq. Step Test | 318.426 | 4.000 | 328.053 |
| Appendicular Lean Mass | Handgrip Strength / BMI | Four Sq. Step Test | 310.841 | 4.000 | 320.418 |
| Appendicular Lean Mass / Ht^2^ | Handgrip Strength / BMI | Four Sq. Step Test | 319.882 | 4.000 | 329.508 |
| Appendicular Lean Mass / BW | Handgrip Strength / BMI | Four Sq. Step Test | 313.583 | 4.000 | 323.160 |
| Appendicular Lean Mass / BMI | Handgrip Strength / BMI | Four Sq. Step Test | 316.140 | 4.000 | 325.718 |

AIC = akaike info criterion; BMI = body mass index; BW = body weight; Ht = height; MPC = mallows’ prediction criterion; SBC = schwarz bayesian criterion; sq = square

| Lower Limb Muscle Mass | Handgrip Strength | Four Sq. Step Test | 323.375 | 4.000 | 333.002 |
| --- | --- | --- | --- | --- | --- |
| Total Muscle Mass | Handgrip Strength | Four Sq. Step Test | 325.125 | 4.000 | 334.752 |
| Appendicular Lean Mass | Handgrip Strength | Four Sq. Step Test | 316.226 | 4.000 | 325.804 |
| Appendicular Lean Mass / Ht^2^ | Handgrip Strength | Four Sq. Step Test | 324.444 | 4.000 | 334.071 |
| Appendicular Lean Mass / BW | Handgrip Strength | Four Sq. Step Test | 312.004 | 4.000 | 321.582 |
| Appendicular Lean Mass / BMI | Handgrip Strength | Four Sq. Step Test | 314.416 | 4.000 | 323.994 |
