# Supplementary material for: Relative contribution of muscle strength, lean mass, and lower extremity motor function in explaining between-person variance in mobility in older adults
Source: BMC Geriatr. 2020 Jul 28;20:255. doi: 10.1186/s12877-020-01656-y (PMC7385889; doi:10.1186/s12877-020-01656-y)
Supplement: Supplementary file 3 — Additional file 3: Table 3. Model of best fit selection for sex (males). [file 12877_2020_1656_MOESM3_ESM.docx]

| **DEPENDENT VARIBLE – 6-MIN WALK GAIT SPEED** | | | | | |
| --- | --- | --- | --- | --- | --- |
| **INDEPENDENT VARIABLES** | | | **SELECTION CRITERIA** | | |
| **Muscle Mass** | **Muscle Strength** | **Motor Function** | **AIC** | **MPC** | **SBC** |
| Lower Limb Muscle Mass | Isokinetic Strength / BW | Four Sq. Step Test | 234.232 | 4.000 | 239.415 |
| Total Muscle Mass | Isokinetic Strength / BW | Four Sq. Step Test | 234.109 | 4.000 | 239.293 |
| Appendicular Lean Mass | Isokinetic Strength / BW | Four Sq. Step Test | 234.298 | 4.000 | 239.481 |
| Appendicular Lean Mass / Ht^2^ | Isokinetic Strength / BW | Four Sq. Step Test | 232.912 | 4.000 | 238.096 |
| Appendicular Lean Mass / BW | Isokinetic Strength / BW | Four Sq. Step Test | 229.902 | 4.000 | 235.085 |
| Appendicular Lean Mass / BMI | Isokinetic Strength / BW | Four Sq. Step Test | 233.954 | 4.000 | 239.138 |
|  |  |  |  |  |  |
| Lower Limb Muscle Mass | Isokinetic Strength / BMI | Four Sq. Step Test | 236.524 | 4.000 | 241.707 |
| Total Muscle Mass | Isokinetic Strength / BMI | Four Sq. Step Test | 235.933 | 4.000 | 241.166 |
| Appendicular Lean Mass | Isokinetic Strength / BMI | Four Sq. Step Test | 236.510 | 4.000 | 241.693 |
| Appendicular Lean Mass / Ht^2^ | Isokinetic Strength / BMI | Four Sq. Step Test | 234.799 | 4.000 | 238.983 |
| Appendicular Lean Mass / BW | Isokinetic Strength / BMI | Four Sq. Step Test | 230.068 | 4.000 | 235.251 |
| Appendicular Lean Mass / BMI | Isokinetic Strength / BMI | Four Sq. Step Test | 235.998 | 4.000 | 241.182 |
|  |  |  |  |  |  |
| Lower Limb Muscle Mass | Isokinetic Strength | Four Sq. Step Test | 238.520 | 4.000 | 243.704 |
| Total Muscle Mass | Isokinetic Strength | Four Sq. Step Test | 236.867 | 4.000 | 242.050 |
| Appendicular Lean Mass | Isokinetic Strength | Four Sq. Step Test | 238.384 | 4.000 | 243.567 |
| Appendicular Lean Mass / Ht^2^ | Isokinetic Strength | Four Sq. Step Test | 237.597 | 4.000 | 242.780 |
| Appendicular Lean Mass / BW | Isokinetic Strength | Four Sq. Step Test | 229.850 | 4.000 | 235.034 |
| Appendicular Lean Mass / BMI | Isokinetic Strength | Four Sq. Step Test | 236.337 | 4.000 | 241.521 |
|  |  |  |  |  |  |
| Lower Limb Muscle Mass | Isometric Strength / BW | Four Sq. Step Test | 238.072 | 4.000 | 243.255 |
| Total Muscle Mass | Isometric Strength / BW | Four Sq. Step Test | 238.716 | 4.000 | 243.900 |
| Appendicular Lean Mass | Isometric Strength / BW | Four Sq. Step Test | 238.343 | 4.000 | 243.526 |
| Appendicular Lean Mass / Ht^2^ | Isometric Strength / BW | Four Sq. Step Test | 236.233 | 4.000 | 241.416 |
| Appendicular Lean Mass / BW | Isometric Strength / BW | Four Sq. Step Test | 229.650 | 4.000 | 234.833 |
| Appendicular Lean Mass / BMI | Isometric Strength / BW | Four Sq. Step Test | 236.366 | 4.000 | 241.550 |
|  |  |  |  |  |  |
| Lower Limb Muscle Mass | Isometric Strength / BMI | Four Sq. Step Test | 238.830 | 4.000 | 244.013 |
| Total Muscle Mass | Isometric Strength / BMI | Four Sq. Step Test | 239.115 | 4.000 | 244.298 |
| Appendicular Lean Mass | Isometric Strength / BMI | Four Sq. Step Test | 239.05 | 4.000 | 244.233 |
| Appendicular Lean Mass / Ht^2^ | Isometric Strength / BMI | Four Sq. Step Test | 236.819 | 4.000 | 242.002 |
| Appendicular Lean Mass / BW | Isometric Strength / BMI | Four Sq. Step Test | 229.362 | 4.000 | 234.545 |
| Appendicular Lean Mass / BMI | Isometric Strength / BMI | Four Sq. Step Test | 236.370 | 4.000 | 241.554 |

| Lower Limb Muscle Mass | Isometric Strength | Four Sq. Step Test | 239.268 | 4.000 | 244.451 |
| --- | --- | --- | --- | --- | --- |
| Total Muscle Mass | Isometric Strength | Four Sq. Step Test | 239.717 | 4.000 | 244.900 |
| Appendicular Lean Mass | Isometric Strength | Four Sq. Step Test | 239.561 | 4.000 | 244.745 |
| Appendicular Lean Mass / Ht^2^ | Isometric Strength | Four Sq. Step Test | 237.911 | 4.000 | 243.095 |
| Appendicular Lean Mass / BW | Isometric Strength | Four Sq. Step Test | 229.834 | 4.000 | 235.017 |
| Appendicular Lean Mass / BMI | Isometric Strength | Four Sq. Step Test | 236.084 | 4.000 | 241.267 |
|  |  |  |  |  |  |
| Lower Limb Muscle Mass | Handgrip Strength / BW | Four Sq. Step Test | 237.491 | 4.000 | 242.675 |
| Total Muscle Mass | Handgrip Strength / BW | Four Sq. Step Test | 238.497 | 4.000 | 243.680 |
| Appendicular Lean Mass | Handgrip Strength / BW | Four Sq. Step Test | 237.907 | 4.000 | 243.090 |
| Appendicular Lean Mass / Ht^2^ | Handgrip Strength / BW | Four Sq. Step Test | 236.032 | 4.000 | 241.215 |
| Appendicular Lean Mass / BW | Handgrip Strength / BW | Four Sq. Step Test | 230.066 | 4.000 | 235.249 |
| Appendicular Lean Mass / BMI | Handgrip Strength / BW | Four Sq. Step Test | 235.771 | 4.000 | 240.955 |
|  |  |  |  |  |  |
| Lower Limb Muscle Mass | Handgrip Strength / BMI | Four Sq. Step Test | 238.720 | 4.000 | 243.903 |
| Total Muscle Mass | Handgrip Strength / BMI | Four Sq. Step Test | 239.250 | 4.000 | 244.433 |
| Appendicular Lean Mass | Handgrip Strength / BMI | Four Sq. Step Test | 239.024 | 4.000 | 244.207 |
| Appendicular Lean Mass / Ht^2^ | Handgrip Strength / BMI | Four Sq. Step Test | 236.849 | 4.000 | 242.032 |
| Appendicular Lean Mass / BW | Handgrip Strength / BMI | Four Sq. Step Test | 229.813 | 4.000 | 234.996 |
| Appendicular Lean Mass / BMI | Handgrip Strength / BMI | Four Sq. Step Test | 236.490 | 4.000 | 241.673 |
|  |  |  |  |  |  |
| Lower Limb Muscle Mass | Handgrip Strength | Four Sq. Step Test | 239.358 | 4.000 | 244.542 |
| Total Muscle Mass | Handgrip Strength | Four Sq. Step Test | 239.647 | 4.000 | 244.831 |
| Appendicular Lean Mass | Handgrip Strength | Four Sq. Step Test | 239.612 | 4.000 | 244.796 |
| Appendicular Lean Mass / Ht^2^ | Handgrip Strength | Four Sq. Step Test | 237.934 | 4.000 | 243.117 |
| Appendicular Lean Mass / BW | Handgrip Strength | Four Sq. Step Test | 229.897 | 4.000 | 235.080 |
| Appendicular Lean Mass / BMI | Handgrip Strength | Four Sq. Step Test | 236.487 | 4.000 | 241.670 |
|  |  |  |  |  |  |
| **DEPENDENT VARIBLE – STAIR CLIMB POWER** | | | | | |
| Lower Limb Muscle Mass | Isokinetic Strength / BW | Four Sq. Step Test | 193.558 | 4.000 | 198.742 |
| Total Muscle Mass | Isokinetic Strength / BW | Four Sq. Step Test | 196.475 | 4.000 | 201.658 |
| Appendicular Lean Mass | Isokinetic Strength / BW | Four Sq. Step Test | 194.356 | 4.000 | 199.539 |
| Appendicular Lean Mass / Ht^2^ | Isokinetic Strength / BW | Four Sq. Step Test | 194.400 | 4.000 | 199.584 |
| Appendicular Lean Mass / BW | Isokinetic Strength / BW | Four Sq. Step Test | 208.695 | 4.000 | 213.879 |
| Appendicular Lean Mass / BMI | Isokinetic Strength / BW | Four Sq. Step Test | 209.462 | 4.000 | 214.646 |

| Lower Limb Muscle Mass | Isokinetic Strength / BMI | Four Sq. Step Test | 192.113 | 4.000 | 197.296 |
| --- | --- | --- | --- | --- | --- |
| Total Muscle Mass | Isokinetic Strength / BMI | Four Sq. Step Test | 196.093 | 4.000 | 201.277 |
| Appendicular Lean Mass | Isokinetic Strength / BMI | Four Sq. Step Test | 192.936 | 4.000 | 198.120 |
| Appendicular Lean Mass / Ht^2^ | Isokinetic Strength / BMI | Four Sq. Step Test | 194.859 | 4.000 | 200.042 |
| Appendicular Lean Mass / BW | Isokinetic Strength / BMI | Four Sq. Step Test | 208.542 | 4.000 | 213.725 |
| Appendicular Lean Mass / BMI | Isokinetic Strength / BMI | Four Sq. Step Test | 209.297 | 4.000 | 214.480 |
| Lower Limb Muscle Mass | Isokinetic Strength / BMI | Four Sq. Step Test | 192.113 | 4.000 | 197.296 |
|  |  |  |  |  |  |
| Lower Limb Muscle Mass | Isokinetic Strength | Four Sq. Step Test | 194.089 | 4.000 | 199.273 |
| Total Muscle Mass | Isokinetic Strength | Four Sq. Step Test | 196.467 | 4.000 | 201.651 |
| Appendicular Lean Mass | Isokinetic Strength | Four Sq. Step Test | 194.803 | 4.000 | 199.986 |
| Appendicular Lean Mass / Ht^2^ | Isokinetic Strength | Four Sq. Step Test | 194.646 | 4.000 | 199.829 |
| Appendicular Lean Mass / BW | Isokinetic Strength | Four Sq. Step Test | 203.839 | 4.000 | 209.022 |
| Appendicular Lean Mass / BMI | Isokinetic Strength | Four Sq. Step Test | 203.896 | 4.000 | 209.080 |
|  |  |  |  |  |  |
| Lower Limb Muscle Mass | Isometric Strength / BW | Four Sq. Step Test | 194.153 | 4.000 | 199.336 |
| Total Muscle Mass | Isometric Strength / BW | Four Sq. Step Test | 196.473 | 4.000 | 201.657 |
| Appendicular Lean Mass | Isometric Strength / BW | Four Sq. Step Test | 194.871 | 4.000 | 200.055 |
| Appendicular Lean Mass / Ht^2^ | Isometric Strength / BW | Four Sq. Step Test | 194.695 | 4.000 | 199.878 |
| Appendicular Lean Mass / BW | Isometric Strength / BW | Four Sq. Step Test | 208.764 | 4.000 | 213.948 |
| Appendicular Lean Mass / BMI | Isometric Strength / BW | Four Sq. Step Test | 208.841 | 4.000 | 214.024 |
|  |  |  |  |  |  |
| Lower Limb Muscle Mass | Isometric Strength / BMI | Four Sq. Step Test | 193.334 | 4.000 | 198.518 |
| Total Muscle Mass | Isometric Strength / BMI | Four Sq. Step Test | 196.157 | 4.000 | 201.341 |
| Appendicular Lean Mass | Isometric Strength / BMI | Four Sq. Step Test | 194.079 | 4.000 | 199.262 |
| Appendicular Lean Mass / Ht^2^ | Isometric Strength / BMI | Four Sq. Step Test | 194.901 | 4.000 | 200.085 |
| Appendicular Lean Mass / BW | Isometric Strength / BMI | Four Sq. Step Test | 208.988 | 4.000 | 214.171 |
| Appendicular Lean Mass / BMI | Isometric Strength / BMI | Four Sq. Step Test | 209.209 | 4.000 | 214.393 |
|  |  |  |  |  |  |
| Lower Limb Muscle Mass | Isometric Strength | Four Sq. Step Test | 194.524 | 4.000 | 199.707 |
| Total Muscle Mass | Isometric Strength | Four Sq. Step Test | 196.485 | 4.000 | 201.668 |
| Appendicular Lean Mass | Isometric Strength | Four Sq. Step Test | 195.155 | 4.000 | 200.338 |
| Appendicular Lean Mass / Ht^2^ | Isometric Strength | Four Sq. Step Test | 194.502 | 4.000 | 199.685 |
| Appendicular Lean Mass / BW | Isometric Strength | Four Sq. Step Test | 208.054 | 4.000 | 213.238 |
| Appendicular Lean Mass / BMI | Isometric Strength | Four Sq. Step Test | 208.468 | 4.000 | 213.651 |

| Lower Limb Muscle Mass | Handgrip Strength / BW | Four Sq. Step Test | 194.537 | 4.000 | 199.721 |
| --- | --- | --- | --- | --- | --- |
| Total Muscle Mass | Handgrip Strength / BW | Four Sq. Step Test | 196.485 | 4.000 | 201.668 |
| Appendicular Lean Mass | Handgrip Strength / BW | Four Sq. Step Test | 195.086 | 4.000 | 200.269 |
| Appendicular Lean Mass / Ht^2^ | Handgrip Strength / BW | Four Sq. Step Test | 194.326 | 4.000 | 199.509 |
| Appendicular Lean Mass / BW | Handgrip Strength / BW | Four Sq. Step Test | 208.471 | 4.000 | 213.655 |
| Appendicular Lean Mass / BMI | Handgrip Strength / BW | Four Sq. Step Test | 208.594 | 4.000 | 213.777 |
|  |  |  |  |  |  |
| Lower Limb Muscle Mass | Handgrip Strength / BMI | Four Sq. Step Test | 193.638 | 4.000 | 198.821 |
| Total Muscle Mass | Handgrip Strength / BMI | Four Sq. Step Test | 195.969 | 4.000 | 201.152 |
| Appendicular Lean Mass | Handgrip Strength / BMI | Four Sq. Step Test | 194.024 | 4.000 | 199.207 |
| Appendicular Lean Mass / Ht^2^ | Handgrip Strength / BMI | Four Sq. Step Test | 194.640 | 4.000 | 199.823 |
| Appendicular Lean Mass / BW | Handgrip Strength / BMI | Four Sq. Step Test | 208.348 | 4.000 | 213.531 |
| Appendicular Lean Mass / BMI | Handgrip Strength / BMI | Four Sq. Step Test | 208.394 | 4.000 | 213.578 |
|  |  |  |  |  |  |
| Lower Limb Muscle Mass | Handgrip Strength | Four Sq. Step Test | 194.577 | 4.000 | 199.761 |
| Total Muscle Mass | Handgrip Strength | Four Sq. Step Test | 196.419 | 4.000 | 201.602 |
| Appendicular Lean Mass | Handgrip Strength | Four Sq. Step Test | 195.220 | 4.000 | 200.403 |
| Appendicular Lean Mass / Ht^2^ | Handgrip Strength | Four Sq. Step Test | 194.618 | 4.000 | 199.801 |
| Appendicular Lean Mass / BW | Handgrip Strength | Four Sq. Step Test | 207.808 | 4.000 | 212.992 |
| Appendicular Lean Mass / BMI | Handgrip Strength | Four Sq. Step Test | 208.118 | 4.000 | 213.302 |
|  |  |  |  |  |  |
| **DEPENDENT VARIBLE – 5x CHAIR RISE TIME** | |  | | | |
| Lower Limb Muscle Mass | Isokinetic Strength / BW | Four Sq. Step Test | 45.089 | 4.000 | 50.273 |
| Total Muscle Mass | Isokinetic Strength / BW | Four Sq. Step Test | 45.109 | 4.000 | 50.292 |
| Appendicular Lean Mass | Isokinetic Strength / BW | Four Sq. Step Test | 45.121 | 4.000 | 50.304 |
| Appendicular Lean Mass / Ht^2^ | Isokinetic Strength / BW | Four Sq. Step Test | 44.545 | 4.000 | 49.729 |
| Appendicular Lean Mass / BW | Isokinetic Strength / BW | Four Sq. Step Test | 45.160 | 4.000 | 50.343 |
| Appendicular Lean Mass / BMI | Isokinetic Strength / BW | Four Sq. Step Test | 45.329 | 4.000 | 50.513 |
|  |  |  |  |  |  |
| Lower Limb Muscle Mass | Isokinetic Strength / BMI | Four Sq. Step Test | 46.627 | 4.000 | 51.811 |
| Total Muscle Mass | Isokinetic Strength / BMI | Four Sq. Step Test | 46.649 | 4.000 | 51.833 |
| Appendicular Lean Mass | Isokinetic Strength / BMI | Four Sq. Step Test | 46.643 | 4.000 | 51.826 |
| Appendicular Lean Mass / Ht^2^ | Isokinetic Strength / BMI | Four Sq. Step Test | 45.527 | 4.000 | 50.711 |
| Appendicular Lean Mass / BW | Isokinetic Strength / BMI | Four Sq. Step Test | 45.967 | 4.000 | 51.150 |
| Appendicular Lean Mass / BMI | Isokinetic Strength / BMI | Four Sq. Step Test | 46.415 | 4.000 | 51.598 |

| Lower Limb Muscle Mass | Isokinetic Strength | Four Sq. Step Test | 47.036 | 4.000 | 52.219 |
| --- | --- | --- | --- | --- | --- |
| Total Muscle Mass | Isokinetic Strength | Four Sq. Step Test | 46.555 | 4.000 | 51.738 |
| Appendicular Lean Mass | Isokinetic Strength | Four Sq. Step Test | 46.884 | 4.000 | 52.067 |
| Appendicular Lean Mass / Ht^2^ | Isokinetic Strength | Four Sq. Step Test | 47.592 | 4.000 | 52.775 |
| Appendicular Lean Mass / BW | Isokinetic Strength | Four Sq. Step Test | 44.713 | 4.000 | 49.896 |
| Appendicular Lean Mass / BMI | Isokinetic Strength | Four Sq. Step Test | 47.269 | 4.000 | 52.452 |
|  |  |  |  |  |  |
| Lower Limb Muscle Mass | Isometric Strength / BW | Four Sq. Step Test | 36.773 | 4.000 | 41.956 |
| Total Muscle Mass | Isometric Strength / BW | Four Sq. Step Test | 36.905 | 4.000 | 42.088 |
| Appendicular Lean Mass | Isometric Strength / BW | Four Sq. Step Test | 36.746 | 4.000 | 41.930 |
| Appendicular Lean Mass / Ht^2^ | Isometric Strength / BW | Four Sq. Step Test | 34.900 | 4.000 | 40.084 |
| Appendicular Lean Mass / BW | Isometric Strength / BW | Four Sq. Step Test | 39.109 | 4.000 | 44.293 |
| Appendicular Lean Mass / BMI | Isometric Strength / BW | Four Sq. Step Test | 39.331 | 4.000 | 44.514 |
|  |  |  |  |  |  |
| Lower Limb Muscle Mass | Isometric Strength / BMI | Four Sq. Step Test | 39.823 | 4.000 | 45.007 |
| Total Muscle Mass | Isometric Strength / BMI | Four Sq. Step Test | 40.095 | 4.000 | 45.278 |
| Appendicular Lean Mass | Isometric Strength / BMI | Four Sq. Step Test | 39.896 | 4.000 | 45.080 |
| Appendicular Lean Mass / Ht^2^ | Isometric Strength / BMI | Four Sq. Step Test | 35.812 | 4.000 | 40.996 |
| Appendicular Lean Mass / BW | Isometric Strength / BMI | Four Sq. Step Test | 39.986 | 4.000 | 45.169 |
| Appendicular Lean Mass / BMI | Isometric Strength / BMI | Four Sq. Step Test | 40.192 | 4.000 | 45.376 |
|  |  |  |  |  |  |
| Lower Limb Muscle Mass | Isometric Strength | Four Sq. Step Test | 41.275 | 4.000 | 46.458 |
| Total Muscle Mass | Isometric Strength | Four Sq. Step Test | 41.008 | 4.000 | 46.191 |
| Appendicular Lean Mass | Isometric Strength | Four Sq. Step Test | 41.262 | 4.000 | 46.445 |
| Appendicular Lean Mass / Ht^2^ | Isometric Strength | Four Sq. Step Test | 40.390 | 4.000 | 45.574 |
| Appendicular Lean Mass / BW | Isometric Strength | Four Sq. Step Test | 36.687 | 4.000 | 41.871 |
| Appendicular Lean Mass / BMI | Isometric Strength | Four Sq. Step Test | 40.746 | 4.000 | 45.929 |
|  |  |  |  |  |  |
| Lower Limb Muscle Mass | Handgrip Strength / BW | Four Sq. Step Test | 45.017 | 4.000 | 50.201 |
| Total Muscle Mass | Handgrip Strength / BW | Four Sq. Step Test | 46.076 | 4.000 | 51.280 |
| Appendicular Lean Mass | Handgrip Strength / BW | Four Sq. Step Test | 45.368 | 4.000 | 50.551 |
| Appendicular Lean Mass / Ht^2^ | Handgrip Strength / BW | Four Sq. Step Test | 45.248 | 4.000 | 50.431 |
| Appendicular Lean Mass / BW | Handgrip Strength / BW | Four Sq. Step Test | 45.491 | 4.000 | 50.675 |
| Appendicular Lean Mass / BMI | Handgrip Strength / BW | Four Sq. Step Test | 46.333 | 4.000 | 51.516 |

| Lower Limb Muscle Mass | Handgrip Strength / BMI | Four Sq. Step Test | 46.597 | 4.000 | 51.780 |
| --- | --- | --- | --- | --- | --- |
| Total Muscle Mass | Handgrip Strength / BMI | Four Sq. Step Test | 47.476 | 4.000 | 52.660 |
| Appendicular Lean Mass | Handgrip Strength / BMI | Four Sq. Step Test | 46.909 | 4.000 | 52.092 |
| Appendicular Lean Mass / Ht^2^ | Handgrip Strength / BMI | Four Sq. Step Test | 45.174 | 4.000 | 50.357 |
| Appendicular Lean Mass / BW | Handgrip Strength / BMI | Four Sq. Step Test | 45.925 | 4.000 | 51.109 |
| Appendicular Lean Mass / BMI | Handgrip Strength / BMI | Four Sq. Step Test | 47.575 | 4.000 | 52.759 |
|  |  |  |  |  |  |
| Lower Limb Muscle Mass | Handgrip Strength | Four Sq. Step Test | 47.958 | 4.000 | 53.141 |
| Total Muscle Mass | Handgrip Strength | Four Sq. Step Test | 48.124 | 4.000 | 53.307 |
| Appendicular Lean Mass | Handgrip Strength | Four Sq. Step Test | 48.069 | 4.000 | 53.252 |
| Appendicular Lean Mass / Ht^2^ | Handgrip Strength | Four Sq. Step Test | 47.584 | 4.000 | 52.768 |
| Appendicular Lean Mass / BW | Handgrip Strength | Four Sq. Step Test | 41.884 | 4.000 | 47.068 |
| Appendicular Lean Mass / BMI | Handgrip Strength | Four Sq. Step Test | 45.905 | 4.000 | 51.089 |
|  |  |  |  |  |  |
| **DEPENDENT VARIBLE – COMPLEX FUNCTION TEST** | |  | | | |
| Lower Limb Muscle Mass | Isokinetic Strength / BW | Four Sq. Step Test | 116.412 | 4.000 | 121.595 |
| Total Muscle Mass | Isokinetic Strength / BW | Four Sq. Step Test | 114.929 | 4.000 | 120.112 |
| Appendicular Lean Mass | Isokinetic Strength / BW | Four Sq. Step Test | 116.244 | 4.000 | 121.428 |
| Appendicular Lean Mass / Ht^2^ | Isokinetic Strength / BW | Four Sq. Step Test | 116.348 | 4.000 | 121.531 |
| Appendicular Lean Mass / BW | Isokinetic Strength / BW | Four Sq. Step Test | 109.490 | 4.000 | 114.673 |
| Appendicular Lean Mass / BMI | Isokinetic Strength / BW | Four Sq. Step Test | 114.962 | 4.000 | 120.145 |
|  |  |  |  |  |  |
| Lower Limb Muscle Mass | Isokinetic Strength / BMI | Four Sq. Step Test | 116.950 | 4.000 | 122.134 |
| Total Muscle Mass | Isokinetic Strength / BMI | Four Sq. Step Test | 115.013 | 4.000 | 120.197 |
| Appendicular Lean Mass | Isokinetic Strength / BMI | Four Sq. Step Test | 116.707 | 4.000 | 121.890 |
| Appendicular Lean Mass / Ht^2^ | Isokinetic Strength / BMI | Four Sq. Step Test | 116.900 | 4.000 | 122.083 |
| Appendicular Lean Mass / BW | Isokinetic Strength / BMI | Four Sq. Step Test | 109.374 | 4.000 | 114.557 |
| Appendicular Lean Mass / BMI | Isokinetic Strength / BMI | Four Sq. Step Test | 114.836 | 4.000 | 120.019 |
|  |  |  |  |  |  |
| Lower Limb Muscle Mass | Isokinetic Strength | Four Sq. Step Test | 118.228 | 4.000 | 123.412 |
| Total Muscle Mass | Isokinetic Strength | Four Sq. Step Test | 115.902 | 4.000 | 121.085 |
| Appendicular Lean Mass | Isokinetic Strength | Four Sq. Step Test | 118.065 | 4.000 | 123.249 |
| Appendicular Lean Mass / Ht^2^ | Isokinetic Strength | Four Sq. Step Test | 117.798 | 4.000 | 122.981 |
| Appendicular Lean Mass / BW | Isokinetic Strength | Four Sq. Step Test | 109.672 | 4.000 | 114.855 |
| Appendicular Lean Mass / BMI | Isokinetic Strength | Four Sq. Step Test | 114.260 | 4.000 | 119.443 |

| Lower Limb Muscle Mass | Isometric Strength / BW | Four Sq. Step Test | 118.073 | 4.000 | 123.257 |
| --- | --- | --- | --- | --- | --- |
| Total Muscle Mass | Isometric Strength / BW | Four Sq. Step Test | 116.727 | 4.000 | 121.910 |
| Appendicular Lean Mass | Isometric Strength / BW | Four Sq. Step Test | 118.017 | 4.000 | 123.200 |
| Appendicular Lean Mass / Ht^2^ | Isometric Strength / BW | Four Sq. Step Test | 117.587 | 4.000 | 122.770 |
| Appendicular Lean Mass / BW | Isometric Strength / BW | Four Sq. Step Test | 108.559 | 4.000 | 113.743 |
| Appendicular Lean Mass / BMI | Isometric Strength / BW | Four Sq. Step Test | 114.934 | 4.000 | 120.117 |
|  |  |  |  |  |  |
| Lower Limb Muscle Mass | Isometric Strength / BMI | Four Sq. Step Test | 118.196 | 4.000 | 123.380 |
| Total Muscle Mass | Isometric Strength / BMI | Four Sq. Step Test | 116.713 | 4.000 | 121.896 |
| Appendicular Lean Mass | Isometric Strength / BMI | Four Sq. Step Test | 118.116 | 4.000 | 123.299 |
| Appendicular Lean Mass / Ht^2^ | Isometric Strength / BMI | Four Sq. Step Test | 117.749 | 4.000 | 122.932 |
| Appendicular Lean Mass / BW | Isometric Strength / BMI | Four Sq. Step Test | 108.312 | 4.000 | 113.495 |
| Appendicular Lean Mass / BMI | Isometric Strength / BMI | Four Sq. Step Test | 113.979 | 4.000 | 119.163 |
|  |  |  |  |  |  |
| Lower Limb Muscle Mass | Isometric Strength | Four Sq. Step Test | 117.376 | 4.000 | 122.559 |
| Total Muscle Mass | Isometric Strength | Four Sq. Step Test | 116.374 | 4.000 | 121.558 |
| Appendicular Lean Mass | Isometric Strength | Four Sq. Step Test | 117.424 | 4.000 | 122.608 |
| Appendicular Lean Mass / Ht^2^ | Isometric Strength | Four Sq. Step Test | 116.846 | 4.000 | 122.029 |
| Appendicular Lean Mass / BW | Isometric Strength | Four Sq. Step Test | 108.112 | 4.000 | 113.295 |
| Appendicular Lean Mass / BMI | Isometric Strength | Four Sq. Step Test | 112.440 | 4.000 | 117.623 |
|  |  |  |  |  |  |
| Lower Limb Muscle Mass | Handgrip Strength / BW | Four Sq. Step Test | 118.150 | 4.000 | 123.333 |
| Total Muscle Mass | Handgrip Strength / BW | Four Sq. Step Test | 116.756 | 4.000 | 121.940 |
| Appendicular Lean Mass | Handgrip Strength / BW | Four Sq. Step Test | 118.099 | 4.000 | 123.283 |
| Appendicular Lean Mass / Ht^2^ | Handgrip Strength / BW | Four Sq. Step Test | 117.694 | 4.000 | 122.877 |
| Appendicular Lean Mass / BW | Handgrip Strength / BW | Four Sq. Step Test | 109.564 | 4.000 | 114.747 |
| Appendicular Lean Mass / BMI | Handgrip Strength / BW | Four Sq. Step Test | 115.055 | 4.000 | 120.238 |
|  |  |  |  |  |  |
| Lower Limb Muscle Mass | Handgrip Strength / BMI | Four Sq. Step Test | 118.251 | 4.000 | 123.434 |
| Total Muscle Mass | Handgrip Strength / BMI | Four Sq. Step Test | 116.749 | 4.000 | 121.932 |
| Appendicular Lean Mass | Handgrip Strength / BMI | Four Sq. Step Test | 118.179 | 4.000 | 123.362 |
| Appendicular Lean Mass / Ht^2^ | Handgrip Strength / BMI | Four Sq. Step Test | 117.823 | 4.000 | 123.006 |
| Appendicular Lean Mass / BW | Handgrip Strength / BMI | Four Sq. Step Test | 108.885 | 4.000 | 114.048 |
| Appendicular Lean Mass / BMI | Handgrip Strength / BMI | Four Sq. Step Test | 114.531 | 4.000 | 119.715 |

AIC = akaike info criterion; BMI = body mass index; BW = body weight; Ht = height; MPC = mallows’ prediction criterion; SBC = schwarz bayesian criterion; sq = square

| Lower Limb Muscle Mass | Handgrip Strength | Four Sq. Step Test | 116.792 | 4.000 | 121.975 |
| --- | --- | --- | --- | --- | --- |
| Total Muscle Mass | Handgrip Strength | Four Sq. Step Test | 115.966 | 4.000 | 121.149 |
| Appendicular Lean Mass | Handgrip Strength | Four Sq. Step Test | 116.842 | 4.000 | 122.026 |
| Appendicular Lean Mass / Ht^2^ | Handgrip Strength | Four Sq. Step Test | 115.963 | 4.000 | 121.146 |
| Appendicular Lean Mass / BW | Handgrip Strength | Four Sq. Step Test | 108.921 | 4.000 | 114.104 |
| Appendicular Lean Mass / BMI | Handgrip Strength | Four Sq. Step Test | 113.174 | 4.000 | 118.357 |
