# Supplementary material for: Relative contribution of muscle strength, lean mass, and lower extremity motor function in explaining between-person variance in mobility in older adults
Source: BMC Geriatr. 2020 Jul 28;20:255. doi: 10.1186/s12877-020-01656-y (PMC7385889; doi:10.1186/s12877-020-01656-y)
Supplement: Supplementary file 4 — Additional file 4: Table 4. Model of best fit selection for sex (females). [file 12877_2020_1656_MOESM4_ESM.docx]

| **DEPENDENT VARIBLE – 6-MIN WALK GAIT SPEED** | | | | | |
| --- | --- | --- | --- | --- | --- |
| **INDEPENDENT VARIABLES** | | | **SELECTION CRITERIA** | | |
| **Muscle Mass** | **Muscle Strength** | **Motor Function** | **AIC** | **MPC** | **SBC** |
| Lower Limb Muscle Mass | Isokinetic Strength / BW | Four Sq. Step Test | 447.817 | 4.000 | 455.773 |
| Total Muscle Mass | Isokinetic Strength / BW | Four Sq. Step Test | 450.670 | 4.000 | 458.626 |
| Appendicular Lean Mass | Isokinetic Strength / BW | Four Sq. Step Test | 438.276 | 4.000 | 446.157 |
| Appendicular Lean Mass / Ht^2^ | Isokinetic Strength / BW | Four Sq. Step Test | 451.222 | 4.000 | 459.178 |
| Appendicular Lean Mass / BW | Isokinetic Strength / BW | Four Sq. Step Test | 443.288 | 4.000 | 451.169 |
| Appendicular Lean Mass / BMI | Isokinetic Strength / BW | Four Sq. Step Test | 442.276 | 4.000 | 450.157 |
|  |  |  |  |  |  |
| Lower Limb Muscle Mass | Isokinetic Strength / BMI | Four Sq. Step Test | 447.545 | 4.000 | 455.501 |
| Total Muscle Mass | Isokinetic Strength / BMI | Four Sq. Step Test | 448.781 | 4.000 | 456.737 |
| Appendicular Lean Mass | Isokinetic Strength / BMI | Four Sq. Step Test | 437.779 | 4.000 | 445.66 |
| Appendicular Lean Mass / Ht^2^ | Isokinetic Strength / BMI | Four Sq. Step Test | 448.708 | 4.000 | 456.664 |
| Appendicular Lean Mass / BW | Isokinetic Strength / BMI | Four Sq. Step Test | 440.439 | 4.000 | 448.32 |
| Appendicular Lean Mass / BMI | Isokinetic Strength / BMI | Four Sq. Step Test | 440.442 | 4.000 | 448.323 |
|  |  |  |  |  |  |
| Lower Limb Muscle Mass | Isokinetic Strength | Four Sq. Step Test | 457.524 | 4.000 | 465.480 |
| Total Muscle Mass | Isokinetic Strength | Four Sq. Step Test | 457.063 | 4.000 | 465.019 |
| Appendicular Lean Mass | Isokinetic Strength | Four Sq. Step Test | 447.545 | 4.000 | 455.426 |
| Appendicular Lean Mass / Ht^2^ | Isokinetic Strength | Four Sq. Step Test | 453.898 | 4.000 | 461.854 |
| Appendicular Lean Mass / BW | Isokinetic Strength | Four Sq. Step Test | 444.360 | 4.000 | 452.241 |
| Appendicular Lean Mass / BMI | Isokinetic Strength | Four Sq. Step Test | 442.668 | 4.000 | 450.550 |
|  |  |  |  |  |  |
| Lower Limb Muscle Mass | Isometric Strength / BW | Four Sq. Step Test | 467.407 | 4.000 | 475.363 |
| Total Muscle Mass | Isometric Strength / BW | Four Sq. Step Test | 467.396 | 4.000 | 475.352 |
| Appendicular Lean Mass | Isometric Strength / BW | Four Sq. Step Test | 458.050 | 4.000 | 465.931 |
| Appendicular Lean Mass / Ht^2^ | Isometric Strength / BW | Four Sq. Step Test | 466.947 | 4.000 | 474.903 |
| Appendicular Lean Mass / BW | Isometric Strength / BW | Four Sq. Step Test | 459.038 | 4.000 | 466.919 |
| Appendicular Lean Mass / BMI | Isometric Strength / BW | Four Sq. Step Test | 454.729 | 4.000 | 462.610 |
|  |  |  |  |  |  |
| Lower Limb Muscle Mass | Isometric Strength / BMI | Four Sq. Step Test | 465.644 | 4.000 | 473.600 |
| Total Muscle Mass | Isometric Strength / BMI | Four Sq. Step Test | 464.887 | 4.000 | 472.843 |
| Appendicular Lean Mass | Isometric Strength / BMI | Four Sq. Step Test | 456.299 | 4.000 | 464.180 |
| Appendicular Lean Mass / Ht^2^ | Isometric Strength / BMI | Four Sq. Step Test | 465.101 | 4.000 | 473.057 |
| Appendicular Lean Mass / BW | Isometric Strength / BMI | Four Sq. Step Test | 456.944 | 4.000 | 464.825 |
| Appendicular Lean Mass / BMI | Isometric Strength / BMI | Four Sq. Step Test | 454.265 | 4.000 | 462.147 |

| Lower Limb Muscle Mass | Isometric Strength | Four Sq. Step Test | 470.023 | 4.000 | 477.979 |
| --- | --- | --- | --- | --- | --- |
| Total Muscle Mass | Isometric Strength | Four Sq. Step Test | 466.927 | 4.000 | 474.883 |
| Appendicular Lean Mass | Isometric Strength | Four Sq. Step Test | 460.988 | 4.000 | 468.869 |
| Appendicular Lean Mass / Ht^2^ | Isometric Strength | Four Sq. Step Test | 466.404 | 4.000 | 474.360 |
| Appendicular Lean Mass / BW | Isometric Strength | Four Sq. Step Test | 458.468 | 4.000 | 466.349 |
| Appendicular Lean Mass / BMI | Isometric Strength | Four Sq. Step Test | 454.425 | 4.000 | 462.306 |
|  |  |  |  |  |  |
| Lower Limb Muscle Mass | Handgrip Strength / BW | Four Sq. Step Test | 470.340 | 4.000 | 478.370 |
| Total Muscle Mass | Handgrip Strength / BW | Four Sq. Step Test | 471.230 | 4.000 | 479.259 |
| Appendicular Lean Mass | Handgrip Strength / BW | Four Sq. Step Test | 460.556 | 4.000 | 468.512 |
| Appendicular Lean Mass / Ht^2^ | Handgrip Strength / BW | Four Sq. Step Test | 471.105 | 4.000 | 479.135 |
| Appendicular Lean Mass / BW | Handgrip Strength / BW | Four Sq. Step Test | 462.866 | 4.000 | 470.822 |
| Appendicular Lean Mass / BMI | Handgrip Strength / BW | Four Sq. Step Test | 460.783 | 4.000 | 468.739 |
|  |  |  |  |  |  |
| Lower Limb Muscle Mass | Handgrip Strength / BMI | Four Sq. Step Test | 470.318 | 4.000 | 478.347 |
| Total Muscle Mass | Handgrip Strength / BMI | Four Sq. Step Test | 470.565 | 4.000 | 478.595 |
| Appendicular Lean Mass | Handgrip Strength / BMI | Four Sq. Step Test | 460.315 | 4.000 | 468.271 |
| Appendicular Lean Mass / Ht^2^ | Handgrip Strength / BMI | Four Sq. Step Test | 470.544 | 4.000 | 478.573 |
| Appendicular Lean Mass / BW | Handgrip Strength / BMI | Four Sq. Step Test | 461.820 | 4.000 | 469.776 |
| Appendicular Lean Mass / BMI | Handgrip Strength / BMI | Four Sq. Step Test | 460.719 | 4.000 | 468.675 |
|  |  |  |  |  |  |
| Lower Limb Muscle Mass | Handgrip Strength | Four Sq. Step Test | 473.531 | 4.000 | 481.56 |
| Total Muscle Mass | Handgrip Strength | Four Sq. Step Test | 472.863 | 4.000 | 480.892 |
| Appendicular Lean Mass | Handgrip Strength | Four Sq. Step Test | 462.956 | 4.000 | 470.912 |
| Appendicular Lean Mass / Ht^2^ | Handgrip Strength | Four Sq. Step Test | 469.973 | 4.000 | 478.002 |
| Appendicular Lean Mass / BW | Handgrip Strength | Four Sq. Step Test | 461.797 | 4.000 | 469.753 |
| Appendicular Lean Mass / BMI | Handgrip Strength | Four Sq. Step Test | 459.750 | 4.000 | 467.706 |
|  |  |  |  |  |  |
| **DEPENDENT VARIBLE – STAIR CLIMB POWER** | | | | | |
| Lower Limb Muscle Mass | Isokinetic Strength / BW | Four Sq. Step Test | 364.102 | 4.000 | 372.058 |
| Total Muscle Mass | Isokinetic Strength / BW | Four Sq. Step Test | 377.022 | 4.000 | 384.978 |
| Appendicular Lean Mass | Isokinetic Strength / BW | Four Sq. Step Test | 357.839 | 4.000 | 365.720 |
| Appendicular Lean Mass / Ht^2^ | Isokinetic Strength / BW | Four Sq. Step Test | 370.955 | 4.000 | 378.911 |
| Appendicular Lean Mass / BW | Isokinetic Strength / BW | Four Sq. Step Test | 374.915 | 4.000 | 382.796 |
| Appendicular Lean Mass / BMI | Isokinetic Strength / BW | Four Sq. Step Test | 378.865 | 4.000 | 386.746 |

| Lower Limb Muscle Mass | Isokinetic Strength / BMI | Four Sq. Step Test | 364.478 | 4.000 | 372.434 |
| --- | --- | --- | --- | --- | --- |
| Total Muscle Mass | Isokinetic Strength / BMI | Four Sq. Step Test | 376.875 | 4.000 | 384.830 |
| Appendicular Lean Mass | Isokinetic Strength / BMI | Four Sq. Step Test | 358.246 | 4.000 | 366.127 |
| Appendicular Lean Mass / Ht^2^ | Isokinetic Strength / BMI | Four Sq. Step Test | 368.997 | 4.000 | 376.953 |
| Appendicular Lean Mass / BW | Isokinetic Strength / BMI | Four Sq. Step Test | 374.189 | 4.000 | 382.070 |
| Appendicular Lean Mass / BMI | Isokinetic Strength / BMI | Four Sq. Step Test | 378.202 | 4.000 | 386.083 |
| Lower Limb Muscle Mass | Isokinetic Strength / BMI | Four Sq. Step Test | 364.478 | 4.000 | 372.434 |
|  |  |  |  |  |  |
| Lower Limb Muscle Mass | Isokinetic Strength | Four Sq. Step Test | 354.659 | 4.000 | 362.615 |
| Total Muscle Mass | Isokinetic Strength | Four Sq. Step Test | 363.602 | 4.000 | 371.558 |
| Appendicular Lean Mass | Isokinetic Strength | Four Sq. Step Test | 349.201 | 4.000 | 357.083 |
| Appendicular Lean Mass / Ht^2^ | Isokinetic Strength | Four Sq. Step Test | 355.920 | 4.000 | 363.876 |
| Appendicular Lean Mass / BW | Isokinetic Strength | Four Sq. Step Test | 357.896 | 4.000 | 365.777 |
| Appendicular Lean Mass / BMI | Isokinetic Strength | Four Sq. Step Test | 359.752 | 4.000 | 367.633 |
|  |  |  |  |  |  |
| Lower Limb Muscle Mass | Isometric Strength / BW | Four Sq. Step Test | 359.795 | 4.000 | 367.750 |
| Total Muscle Mass | Isometric Strength / BW | Four Sq. Step Test | 373.913 | 4.000 | 381.869 |
| Appendicular Lean Mass | Isometric Strength / BW | Four Sq. Step Test | 352.133 | 4.000 | 360.014 |
| Appendicular Lean Mass / Ht^2^ | Isometric Strength / BW | Four Sq. Step Test | 371.167 | 4.000 | 379.123 |
| Appendicular Lean Mass / BW | Isometric Strength / BW | Four Sq. Step Test | 375.538 | 4.000 | 383.419 |
| Appendicular Lean Mass / BMI | Isometric Strength / BW | Four Sq. Step Test | 378.937 | 4.000 | 386.818 |
|  |  |  |  |  |  |
| Lower Limb Muscle Mass | Isometric Strength / BMI | Four Sq. Step Test | 359.802 | 4.000 | 367.758 |
| Total Muscle Mass | Isometric Strength / BMI | Four Sq. Step Test | 374.337 | 4.000 | 382.293 |
| Appendicular Lean Mass | Isometric Strength / BMI | Four Sq. Step Test | 352.131 | 4.000 | 360.012 |
| Appendicular Lean Mass / Ht^2^ | Isometric Strength / BMI | Four Sq. Step Test | 370.984 | 4.000 | 378.940 |
| Appendicular Lean Mass / BW | Isometric Strength / BMI | Four Sq. Step Test | 375.120 | 4.000 | 383.001 |
| Appendicular Lean Mass / BMI | Isometric Strength / BMI | Four Sq. Step Test | 379.367 | 4.000 | 387.248 |
|  |  |  |  |  |  |
| Lower Limb Muscle Mass | Isometric Strength | Four Sq. Step Test | 357.606 | 4.000 | 365.562 |
| Total Muscle Mass | Isometric Strength | Four Sq. Step Test | 374.478 | 4.000 | 382.434 |
| Appendicular Lean Mass | Isometric Strength | Four Sq. Step Test | 350.480 | 4.000 | 358.362 |
| Appendicular Lean Mass / Ht^2^ | Isometric Strength | Four Sq. Step Test | 366.603 | 4.000 | 374.559 |
| Appendicular Lean Mass / BW | Isometric Strength | Four Sq. Step Test | 364.786 | 4.000 | 372.667 |
| Appendicular Lean Mass / BMI | Isometric Strength | Four Sq. Step Test | 370.264 | 4.000 | 378.145 |

| Lower Limb Muscle Mass | Handgrip Strength / BW | Four Sq. Step Test | 374.163 | 4.000 | 382.192 |
| --- | --- | --- | --- | --- | --- |
| Total Muscle Mass | Handgrip Strength / BW | Four Sq. Step Test | 384.421 | 4.000 | 392.451 |
| Appendicular Lean Mass | Handgrip Strength / BW | Four Sq. Step Test | 367.450 | 4.000 | 375.405 |
| Appendicular Lean Mass / Ht^2^ | Handgrip Strength / BW | Four Sq. Step Test | 381.971 | 4.000 | 390.000 |
| Appendicular Lean Mass / BW | Handgrip Strength / BW | Four Sq. Step Test | 383.374 | 4.000 | 391.33 |
| Appendicular Lean Mass / BMI | Handgrip Strength / BW | Four Sq. Step Test | 384.852 | 4.000 | 392.808 |
|  |  |  |  |  |  |
| Lower Limb Muscle Mass | Handgrip Strength / BMI | Four Sq. Step Test | 374.156 | 4.000 | 382.186 |
| Total Muscle Mass | Handgrip Strength / BMI | Four Sq. Step Test | 384.588 | 4.000 | 392.618 |
| Appendicular Lean Mass | Handgrip Strength / BMI | Four Sq. Step Test | 367.410 | 4.000 | 375.366 |
| Appendicular Lean Mass / Ht^2^ | Handgrip Strength / BMI | Four Sq. Step Test | 381.899 | 4.000 | 389.928 |
| Appendicular Lean Mass / BW | Handgrip Strength / BMI | Four Sq. Step Test | 383.393 | 4.000 | 391.349 |
| Appendicular Lean Mass / BMI | Handgrip Strength / BMI | Four Sq. Step Test | 358.647 | 4.000 | 393.603 |
|  |  |  |  |  |  |
| Lower Limb Muscle Mass | Handgrip Strength | Four Sq. Step Test | 371.490 | 4.000 | 379.520 |
| Total Muscle Mass | Handgrip Strength | Four Sq. Step Test | 380.149 | 4.000 | 388.178 |
| Appendicular Lean Mass | Handgrip Strength | Four Sq. Step Test | 365.450 | 4.000 | 373.406 |
| Appendicular Lean Mass / Ht^2^ | Handgrip Strength | Four Sq. Step Test | 375.299 | 4.000 | 383.328 |
| Appendicular Lean Mass / BW | Handgrip Strength | Four Sq. Step Test | 370.702 | 4.000 | 378.658 |
| Appendicular Lean Mass / BMI | Handgrip Strength | Four Sq. Step Test | 373.809 | 4.000 | 381.765 |
|  |  |  |  |  |  |
| **DEPENDENT VARIBLE – 5x CHAIR RISE TIME** | |  | | | |
| Lower Limb Muscle Mass | Isokinetic Strength / BW | Four Sq. Step Test | 143.817 | 4.000 | 151.773 |
| Total Muscle Mass | Isokinetic Strength / BW | Four Sq. Step Test | 143.691 | 4.000 | 151.647 |
| Appendicular Lean Mass | Isokinetic Strength / BW | Four Sq. Step Test | 142.277 | 4.000 | 150.158 |
| Appendicular Lean Mass / Ht^2^ | Isokinetic Strength / BW | Four Sq. Step Test | 143.288 | 4.000 | 151.243 |
| Appendicular Lean Mass / BW | Isokinetic Strength / BW | Four Sq. Step Test | 142.318 | 4.000 | 150.199 |
| Appendicular Lean Mass / BMI | Isokinetic Strength / BW | Four Sq. Step Test | 141.560 | 4.000 | 149.441 |
|  |  |  |  |  |  |
| Lower Limb Muscle Mass | Isokinetic Strength / BMI | Four Sq. Step Test | 146.621 | 4.000 | 154.577 |
| Total Muscle Mass | Isokinetic Strength / BMI | Four Sq. Step Test | 146.692 | 4.000 | 154.648 |
| Appendicular Lean Mass | Isokinetic Strength / BMI | Four Sq. Step Test | 144.876 | 4.000 | 152.757 |
| Appendicular Lean Mass / Ht^2^ | Isokinetic Strength / BMI | Four Sq. Step Test | 146.479 | 4.000 | 154.435 |
| Appendicular Lean Mass / BW | Isokinetic Strength / BMI | Four Sq. Step Test | 144.725 | 4.000 | 152.606 |
| Appendicular Lean Mass / BMI | Isokinetic Strength / BMI | Four Sq. Step Test | 144.131 | 4.000 | 152.013 |

| Lower Limb Muscle Mass | Isokinetic Strength | Four Sq. Step Test | 146.462 | 4.000 | 154.418 |
| --- | --- | --- | --- | --- | --- |
| Total Muscle Mass | Isokinetic Strength | Four Sq. Step Test | 147.793 | 4.000 | 155.749 |
| Appendicular Lean Mass | Isokinetic Strength | Four Sq. Step Test | 144.372 | 4.000 | 152.253 |
| Appendicular Lean Mass / Ht^2^ | Isokinetic Strength | Four Sq. Step Test | 147.944 | 4.000 | 155.900 |
| Appendicular Lean Mass / BW | Isokinetic Strength | Four Sq. Step Test | 144.240 | 4.000 | 152.121 |
| Appendicular Lean Mass / BMI | Isokinetic Strength | Four Sq. Step Test | 146.007 | 4.000 | 153.888 |
|  |  |  |  |  |  |
| Lower Limb Muscle Mass | Isometric Strength / BW | Four Sq. Step Test | 145.622 | 4.000 | 153.578 |
| Total Muscle Mass | Isometric Strength / BW | Four Sq. Step Test | 145.458 | 4.000 | 153.413 |
| Appendicular Lean Mass | Isometric Strength / BW | Four Sq. Step Test | 143.980 | 4.000 | 151.861 |
| Appendicular Lean Mass / Ht^2^ | Isometric Strength / BW | Four Sq. Step Test | 145.669 | 4.000 | 153.624 |
| Appendicular Lean Mass / BW | Isometric Strength / BW | Four Sq. Step Test | 144.043 | 4.000 | 151.924 |
| Appendicular Lean Mass / BMI | Isometric Strength / BW | Four Sq. Step Test | 143.715 | 4.000 | 151.596 |
|  |  |  |  |  |  |
| Lower Limb Muscle Mass | Isometric Strength / BMI | Four Sq. Step Test | 147.330 | 4.000 | 155.286 |
| Total Muscle Mass | Isometric Strength / BMI | Four Sq. Step Test | 147.204 | 4.000 | 155.160 |
| Appendicular Lean Mass | Isometric Strength / BMI | Four Sq. Step Test | 145.577 | 4.000 | 153.459 |
| Appendicular Lean Mass / Ht^2^ | Isometric Strength / BMI | Four Sq. Step Test | 147.638 | 4.000 | 155.594 |
| Appendicular Lean Mass / BW | Isometric Strength / BMI | Four Sq. Step Test | 145.653 | 4.000 | 153.534 |
| Appendicular Lean Mass / BMI | Isometric Strength / BMI | Four Sq. Step Test | 145.419 | 4.000 | 153.300 |
|  |  |  |  |  |  |
| Lower Limb Muscle Mass | Isometric Strength | Four Sq. Step Test | 146.222 | 4.000 | 154.178 |
| Total Muscle Mass | Isometric Strength | Four Sq. Step Test | 145.928 | 4.000 | 153.884 |
| Appendicular Lean Mass | Isometric Strength | Four Sq. Step Test | 144.243 | 4.000 | 152.124 |
| Appendicular Lean Mass / Ht^2^ | Isometric Strength | Four Sq. Step Test | 147.698 | 4.000 | 155.654 |
| Appendicular Lean Mass / BW | Isometric Strength | Four Sq. Step Test | 145.195 | 4.000 | 153.076 |
| Appendicular Lean Mass / BMI | Isometric Strength | Four Sq. Step Test | 146.407 | 4.000 | 154.289 |
|  |  |  |  |  |  |
| Lower Limb Muscle Mass | Handgrip Strength / BW | Four Sq. Step Test | 147.287 | 4.000 | 155.296 |
| Total Muscle Mass | Handgrip Strength / BW | Four Sq. Step Test | 147.238 | 4.000 | 155.268 |
| Appendicular Lean Mass | Handgrip Strength / BW | Four Sq. Step Test | 145.603 | 4.000 | 153.559 |
| Appendicular Lean Mass / Ht^2^ | Handgrip Strength / BW | Four Sq. Step Test | 147.027 | 4.000 | 155.056 |
| Appendicular Lean Mass / BW | Handgrip Strength / BW | Four Sq. Step Test | 145.496 | 4.000 | 153.451 |
| Appendicular Lean Mass / BMI | Handgrip Strength / BW | Four Sq. Step Test | 144.425 | 4.000 | 152.380 |

| Lower Limb Muscle Mass | Handgrip Strength / BMI | Four Sq. Step Test | 149.494 | 4.000 | 157.523 |
| --- | --- | --- | --- | --- | --- |
| Total Muscle Mass | Handgrip Strength / BMI | Four Sq. Step Test | 149.581 | 4.000 | 157.611 |
| Appendicular Lean Mass | Handgrip Strength / BMI | Four Sq. Step Test | 147.684 | 4.000 | 155.639 |
| Appendicular Lean Mass / Ht^2^ | Handgrip Strength / BMI | Four Sq. Step Test | 149.512 | 4.000 | 157.542 |
| Appendicular Lean Mass / BW | Handgrip Strength / BMI | Four Sq. Step Test | 147.613 | 4.000 | 155.569 |
| Appendicular Lean Mass / BMI | Handgrip Strength / BMI | Four Sq. Step Test | 146.739 | 4.000 | 154.695 |
|  |  |  |  |  |  |
| Lower Limb Muscle Mass | Handgrip Strength | Four Sq. Step Test | 148.608 | 4.000 | 156.638 |
| Total Muscle Mass | Handgrip Strength | Four Sq. Step Test | 150.219 | 4.000 | 158.248 |
| Appendicular Lean Mass | Handgrip Strength | Four Sq. Step Test | 146.161 | 4.000 | 154.117 |
| Appendicular Lean Mass / Ht^2^ | Handgrip Strength | Four Sq. Step Test | 150.470 | 4.000 | 158.499 |
| Appendicular Lean Mass / BW | Handgrip Strength | Four Sq. Step Test | 147.336 | 4.000 | 155.292 |
| Appendicular Lean Mass / BMI | Handgrip Strength | Four Sq. Step Test | 148.537 | 4.000 | 156.493 |
|  |  |  |  |  |  |
| **DEPENDENT VARIBLE – COMPLEX FUNCTION TEST** | |  | | | |
| Lower Limb Muscle Mass | Isokinetic Strength / BW | Four Sq. Step Test | 178.899 | 4.000 | 186.855 |
| Total Muscle Mass | Isokinetic Strength / BW | Four Sq. Step Test | 189.951 | 4.000 | 197.907 |
| Appendicular Lean Mass | Isokinetic Strength / BW | Four Sq. Step Test | 174.748 | 4.000 | 182.629 |
| Appendicular Lean Mass / Ht^2^ | Isokinetic Strength / BW | Four Sq. Step Test | 182.172 | 4.000 | 190.128 |
| Appendicular Lean Mass / BW | Isokinetic Strength / BW | Four Sq. Step Test | 190.742 | 4.000 | 198.624 |
| Appendicular Lean Mass / BMI | Isokinetic Strength / BW | Four Sq. Step Test | 190.575 | 4.000 | 198.457 |
|  |  |  |  |  |  |
| Lower Limb Muscle Mass | Isokinetic Strength / BMI | Four Sq. Step Test | 177.311 | 4.000 | 185.266 |
| Total Muscle Mass | Isokinetic Strength / BMI | Four Sq. Step Test | 189.180 | 4.000 | 197.136 |
| Appendicular Lean Mass | Isokinetic Strength / BMI | Four Sq. Step Test | 172.716 | 4.000 | 180.598 |
| Appendicular Lean Mass / Ht^2^ | Isokinetic Strength / BMI | Four Sq. Step Test | 182.990 | 4.000 | 190.946 |
| Appendicular Lean Mass / BW | Isokinetic Strength / BMI | Four Sq. Step Test | 191.676 | 4.000 | 199.557 |
| Appendicular Lean Mass / BMI | Isokinetic Strength / BMI | Four Sq. Step Test | 190.579 | 4.000 | 198.460 |
|  |  |  |  |  |  |
| Lower Limb Muscle Mass | Isokinetic Strength | Four Sq. Step Test | 178.328 | 4.000 | 186.283 |
| Total Muscle Mass | Isokinetic Strength | Four Sq. Step Test | 195.087 | 4.000 | 203.043 |
| Appendicular Lean Mass | Isokinetic Strength | Four Sq. Step Test | 173.654 | 4.000 | 181.535 |
| Appendicular Lean Mass / Ht^2^ | Isokinetic Strength | Four Sq. Step Test | 184.141 | 4.000 | 192.097 |
| Appendicular Lean Mass / BW | Isokinetic Strength | Four Sq. Step Test | 198.227 | 4.000 | 206.108 |
| Appendicular Lean Mass / BMI | Isokinetic Strength | Four Sq. Step Test | 198.910 | 4.000 | 206.791 |

| Lower Limb Muscle Mass | Isometric Strength / BW | Four Sq. Step Test | 177.597 | 4.000 | 185.552 |
| --- | --- | --- | --- | --- | --- |
| Total Muscle Mass | Isometric Strength / BW | Four Sq. Step Test | 185.353 | 4.000 | 193.309 |
| Appendicular Lean Mass | Isometric Strength / BW | Four Sq. Step Test | 173.457 | 4.000 | 181.339 |
| Appendicular Lean Mass / Ht^2^ | Isometric Strength / BW | Four Sq. Step Test | 179.529 | 4.000 | 187.485 |
| Appendicular Lean Mass / BW | Isometric Strength / BW | Four Sq. Step Test | 187.841 | 4.000 | 195.722 |
| Appendicular Lean Mass / BMI | Isometric Strength / BW | Four Sq. Step Test | 187.885 | 4.000 | 195.766 |
|  |  |  |  |  |  |
| Lower Limb Muscle Mass | Isometric Strength / BMI | Four Sq. Step Test | 176.664 | 4.000 | 184.619 |
| Total Muscle Mass | Isometric Strength / BMI | Four Sq. Step Test | 184.363 | 4.000 | 192.319 |
| Appendicular Lean Mass | Isometric Strength / BMI | Four Sq. Step Test | 172.288 | 4.000 | 180.169 |
| Appendicular Lean Mass / Ht^2^ | Isometric Strength / BMI | Four Sq. Step Test | 180.134 | 4.000 | 188.090 |
| Appendicular Lean Mass / BW | Isometric Strength / BMI | Four Sq. Step Test | 187.985 | 4.000 | 195.866 |
| Appendicular Lean Mass / BMI | Isometric Strength / BMI | Four Sq. Step Test | 187.918 | 4.000 | 195.800 |
|  |  |  |  |  |  |
| Lower Limb Muscle Mass | Isometric Strength | Four Sq. Step Test | 177.474 | 4.000 | 185.430 |
| Total Muscle Mass | Isometric Strength | Four Sq. Step Test | 188.654 | 4.000 | 196.610 |
| Appendicular Lean Mass | Isometric Strength | Four Sq. Step Test | 173.301 | 4.000 | 181.182 |
| Appendicular Lean Mass / Ht^2^ | Isometric Strength | Four Sq. Step Test | 180.799 | 4.000 | 188.755 |
| Appendicular Lean Mass / BW | Isometric Strength | Four Sq. Step Test | 190.262 | 4.000 | 198.143 |
| Appendicular Lean Mass / BMI | Isometric Strength | Four Sq. Step Test | 190.230 | 4.000 | 198.111 |
|  |  |  |  |  |  |
| Lower Limb Muscle Mass | Handgrip Strength / BW | Four Sq. Step Test | 181.124 | 4.000 | 189.153 |
| Total Muscle Mass | Handgrip Strength / BW | Four Sq. Step Test | 192.179 | 4.000 | 200.209 |
| Appendicular Lean Mass | Handgrip Strength / BW | Four Sq. Step Test | 176.828 | 4.000 | 184.784 |
| Appendicular Lean Mass / Ht^2^ | Handgrip Strength / BW | Four Sq. Step Test | 184.889 | 4.000 | 192.918 |
| Appendicular Lean Mass / BW | Handgrip Strength / BW | Four Sq. Step Test | 192.446 | 4.000 | 200.402 |
| Appendicular Lean Mass / BMI | Handgrip Strength / BW | Four Sq. Step Test | 192.506 | 4.000 | 200.462 |
|  |  |  |  |  |  |
| Lower Limb Muscle Mass | Handgrip Strength / BMI | Four Sq. Step Test | 180.350 | 4.000 | 188.380 |
| Total Muscle Mass | Handgrip Strength / BMI | Four Sq. Step Test | 191.889 | 4.000 | 199.918 |
| Appendicular Lean Mass | Handgrip Strength / BMI | Four Sq. Step Test | 175.615 | 4.000 | 183.571 |
| Appendicular Lean Mass / Ht^2^ | Handgrip Strength / BMI | Four Sq. Step Test | 186.237 | 4.000 | 194.266 |
| Appendicular Lean Mass / BW | Handgrip Strength / BMI | Four Sq. Step Test | 194.368 | 4.000 | 202.324 |
| Appendicular Lean Mass / BMI | Handgrip Strength / BMI | Four Sq. Step Test | 192.394 | 4.000 | 200.350 |

AIC = akaike info criterion; BMI = body mass index; BW = body weight; Ht = height; MPC = mallows’ prediction criterion; SBC = schwarz bayesian criterion; sq = square

| Lower Limb Muscle Mass | Handgrip Strength | Four Sq. Step Test | 178.518 | 4.000 | 186.547 |
| --- | --- | --- | --- | --- | --- |
| Total Muscle Mass | Handgrip Strength | Four Sq. Step Test | 197.341 | 4.000 | 205.370 |
| Appendicular Lean Mass | Handgrip Strength | Four Sq. Step Test | 172.205 | 4.000 | 180.161 |
| Appendicular Lean Mass / Ht^2^ | Handgrip Strength | Four Sq. Step Test | 186.610 | 4.000 | 194.639 |
| Appendicular Lean Mass / BW | Handgrip Strength | Four Sq. Step Test | 200.994 | 4.000 | 208.950 |
| Appendicular Lean Mass / BMI | Handgrip Strength | Four Sq. Step Test | 201.584 | 4.000 | 209.540 |
